# Weak Acid Permeation in Synthetic Lipid Vesicles and Across the Yeast Plasma Membrane

**DOI:** 10.1016/j.bpj.2019.11.3384

**Published:** 2019-11-27

**Authors:** Matteo Gabba, Jacopo Frallicciardi, Joury van ’t Klooster, Ryan Henderson, Łukasz Syga, Robert Mans, Antonius J.A. van Maris, Bert Poolman

**Affiliations:** 1 Department of Biochemistry, Groningen Biomolecular Sciences and Biotechnology Institute, University of Groningen, Groningen, the Netherlands; 2 Department of Industrial Biotechnology, Delft University of Technology, Delft, the Netherlands; 3 Industrial Biotechnology Division, KTH Royal Institute of Technology, Stockholm, Sweden

## Abstract

We present a fluorescence-based approach for determination of the permeability of small molecules across the membranes of lipid vesicles and living cells. With properly designed experiments, the method allows us to assess the membrane physical properties both in vitro and in vivo. We find that the permeability of weak acids increases in the order of benzoic > acetic > formic > lactic, both in synthetic lipid vesicles and the plasma membrane of *Saccharomyces cerevisiae*, but the permeability is much lower in yeast (one to two orders of magnitude). We observe a relation between the molecule permeability and the saturation of the lipid acyl chain (i.e., lipid packing) in the synthetic lipid vesicles. By analyzing wild-type yeast and a manifold knockout strain lacking all putative lactic acid transporters, we conclude that the yeast plasma membrane is impermeable to lactic acid on timescales up to ∼2.5 h.

## Significance

We present a (stopped-flow) fluorescence-based assay for quantitative determination of the membrane permeability of small molecules both in lipid vesicles and in living cells. The assay provides a measure of the membrane permeability by retrieving permeability coefficients (cm/s). The method can serve the following purposes: 1) to measure the membrane permeability of molecules such as weak acids and bases, glycerol, sugars, and other metabolites; 2) to correlate protein-mediated transport activity to the membrane physical properties; and 3) to relate membrane physical properties to lipid composition and temperature.

## Introduction

Many cellular processes and the robustness of cells to environmental conditions (pH, temperature, osmolality) lean on the biophysical properties of the plasma membrane, which is the lipid bilayer separating the intracellular environment from the external world. These processes include 1) the activity of membrane proteins (transporters, receptors, etc.), which depends on the lipid composition of the membrane; 2) the encounter rate of two molecular partners embedded in the membrane, which depends on their lateral diffusion; and 3) cellular metabolism, which is affected by the passive diffusion across the plasma membrane of some chemical species (ethanol, CO_2_, water, weak acids, etc.). The biophysical properties of the membrane include 1) excluded volume effects, which are caused by membrane crowding and lipid packing; 2) membrane fluidity, which influences the lateral mobility of molecules; 3) lipid phase separation, which can affect the partitioning of membrane proteins; 4) surface charge distribution; and 5) membrane polarization (1, 2, 3). Thus, tight control of the plasma membrane biophysical state is required for proper functioning of the cell.

The permeability of membranes for small molecules depends on their capability to access the free space available between the lipid headgroups and in the hydrocarbon core (4,5). Thus, permeability measurements indirectly report on the membrane physical properties and can assess the impact of these properties on the aforementioned processes both in vivo and in vitro. Besides the characterization of the membrane physical property, the possibility to determine permeability of small molecules in vivo is valuable per se. Weak acids may diffuse into the cell in their neutral form (AH), leading to acidification of the intracellular milieu and growth inhibition (6,7). It is generally believed that this is the major mechanism behind the use of weak acids (benzoic, acetic, sorbic, propionic, and lactic acid) as food preservatives (7); the other mechanism relates to toxicity effects of the corresponding anions on cellular metabolism (8). Moreover, biotechnological production of weak acids is important in the chemical industry (9). For instance, lactic acid can be produced by many microbial species, such as the commercially important *Lactobacillus* strains, (engineered) lactic acid bacteria, fungi, and engineered yeasts (for a recent overview, see (10)). Among the engineered species, the popular yeast *Saccharomyces cerevisiae* has been extensively evaluated for its potential as a lactic acid producer (11, 12, 13). Although transport proteins involved in lactate anion uptake have been described for *S. cerevisiae* (11,14, 15, 16, 17), possible export mechanisms remain enigmatic (18,19), and direct evidence for lactic acid diffusion through the yeast plasma membrane is missing. More generally, the contribution of weak acid passive diffusion and carrier-mediated transport is unclear and difficult to assess. This illustrates the importance of developing an easy method to monitor the diffusion of weak acids across the membranes of living cells.

Here, we first set up a stopped-flow fluorescence-based assay to determine permeability coefficients in vitro. By using the in vitro assay on lipid vesicles prepared with different degree of acyl chain saturation, we detect variations of the permeability as a function of the lipid composition. Then, we extended our approach to an in vivo situation, using *S. cerevisiae* as a model organism, and we benchmarked passive diffusion of weak acids in wild-type yeast against a knockout strain lacking all known and a large set of putative lactic acid transporters. In the accompanying work (20), we describe the modeling of the relaxation dynamics of vesicles and cells exposed to osmotic shifts, which allows one to obtain permeability coefficients from the kinetics of the fluorescence-based assays.

## Materials and Methods

### Materials

The weak acid solutions were prepared using the following salts: sodium benzoate (bio extra ≥99.5%, B3420-250G; Sigma-Aldrich, St. Louis, MO); potassium acetate (extrapure; Merck); sodium formate (pro analysis; Merck, Darmstadt, Germany); DL-lactic acid lithium salt (approximately 98%, L1500; Sigma-Aldrich); pyruvic acid-sodium salt (99+%; Acros Organics, Geel, Belgium); succinic acid-disodium salt, anhydrous (99%; Acros Organics); potassium chloride (pro analyses; BOOM Laboratoriumleveranciers, Meppel, The Netherlands); and sodium L-lactate (>99.0%, 71718-10G; Sigma-Aldrich). Lipids were purchased from Avanti Polar Lipids (Alabaster, AL). The following lipids were used: 1,2-dioleoyl-*sn*-glycero-3-phosphocholine (DOPC), 1,2-dioleoyl-*sn*-glycero-3-phosphoethanolamine (DOPE), 1,2-dioleoyl-*sn*-glycero-3-phospho-(1′-rac-glycerol) sodium salt (DOPG), 1-palmitoyl-2-oleoyl-*sn*-glycero-3-phosphocholine (POPC), 1-palmitoyl-2-oleoyl-*sn*-glycero-3-phosphoethanolamine (POPE), 1-palmitoyl-2-oleoyl-*sn*-glycero-3-phospho-(1′-rac-glycerol) sodium salt (POPG), 1,2-dipalmitoyl-*sn*-glycero-3-phosphocholine (DPPC), 1,2-dipalmitoyl-*sn*-glycero-3-phosphoethanolamine (DPPE), and 1,2-dipalmitoyl-*sn*-glycero-3-phospho-(1′-rac-glycerol) sodium salt (DPPG).

### Weak acid solutions

The 1 M stock solutions (0.5 M for benzoic acid) were prepared by dissolving the salt into 100 mM potassium phosphate (KPi), and the pH was adjusted to 7.0 using 5 M NaOH. For each solution, an empirical linear relation (y = mx + q) between concentration and osmolality was determined (see Fig. S1). The osmolality was measured using a freezing point depression osmometer (Osmomat 030; Genotec, Berlin, Germany). The empirical relations were used to calculate the weak acid concentrations required to obtain an osmolality of ∼300 mosmol/kg, that is, upon mixing with the liposome solution. Accordingly, the stock solutions were diluted to the desired concentration before the experiment. The exceptions are the lactic and pyruvic acid solutions, which, for stability reasons, were freshly prepared at the desired concentration right before the measurement.

### Liposomes preparation

Liposomes were prepared as previously described (21), using six different synthetic lipid mixtures: 1) DOPE:DOPG:DOPC, 2) POPE:POPG:POPC, 3) 67% DOPE:DOPG:DOPC + 33% POPE:POPG:POPC, 4) 33% DOPE:DOPG:DOPC + 67% POPE:POPG:POP, 5) 67% POPE:POPG:POPC + 33% DPPE:DPPG:DPPC, and 6) 33% POPE:POPG:POPC + 67% DPPE:DPPG:DPPC. The lipids (25 mg/mL in chloroform) were purchased from Avanti Polar Lipids and mixed in a 2:1:1 (PE/PG/PC) weight ratio. The exceptions are DPPE and DPPG, which were purchased as powder and dissolved in chloroform/methanol/water (65:35:8) and chloroform/methanol (5:1), respectively. The organic solvents (chloroform, mainly) were removed by evaporation with a rotary vaporizer (Rotovapor r-3; BUCHI, Flawil, Switzerland). Lipids were suspended in diethylether, followed by evaporation, and finally rehydrated in assay buffer (100 mM KPi (pH 7.0)) to a concentration of 10 mg/mL. The liposome solution was homogenized by tip sonication with a Sonics Vibra Cell sonicator (Sonics & Materials, Newton, CT) at 0°C for 30 s with 5 s pulses and 5 s pause between every pulse. The amplitude was set to 100%. Subsequently, the liposomes were snap frozen and thawed at 30°C (65°C for mixtures containing DP lipids) for two times. The prepared liposomes were aliquoted (2 mg/0.2 mL) and stocked in liquid nitrogen to prevent oxidation.

### Preparation of liposomes filled with calcein

The fluorophore calcein (from Sigma-Aldrich) was prepared at a concentration of 100 mM in 50 mM KPi, and the pH was adjusted to 7.0 using 5 M KOH. The stocked liposomes (2 mg of lipid) were pelleted by ultracentrifugation (80,000 rpm, 4°C, 20 min with a TLA 100.3 rotor in a Beckman Optima TLX Ultracentrifuge; Beckman Coulter Life Sciences, Indianapolis, IN) and resuspended in 0.9 mL of 89 mM KPi (pH 7.0). Calcein was added to the liposome solution at a self-quenching concentration (10 mM) and enclosed in the liposomes by three freeze-and-thaw cycles at 30°C (65°C for mixtures containing DPPE, DPPC, and DPPG lipids). Thus, the osmolality of the liposome lumen (filled with 10 mM calcein plus 85 mM KPi (pH 7.0)) is ∼190 mosmol/kg. This value equals the osmolality of the assay buffer (100 mM KPi (pH 7.0)). After extrusion through a 200 nm polycarbonate filter at 20°C (65°C for mixtures containing DPPE, DPPC, and DPPG lipids) to homogenize the vesicles, the liposomes were eluted through a 22-cm-long Sephadex-G75 (Sigma-Aldrich) column pre-equilibrated with the assay buffer to remove the external calcein. The collected 1 mL fractions containing the calcein-filled liposomes were identified by eye using an ultraviolet lamp (for fluorophore excitation) and diluted in a total volume of 12 mL of the assay buffer. To rule out a possible pH dependence of the calcein assay readout, we measured fluorescence emission spectra of free calcein (10 *μ*M) in 100 mM KPi at pH values of 6.0, 6.5, and 7.0 under identical conditions (see Fig. S2). Clearly, the calcein emission spectra at these pH values are the same, and consequently, the assay readout is not affected by the pH.

### Preparation of liposomes filled with pyranine

The ratiometric pH-sensitive fluorophore pyranine (from Molecular Probes, Eugene, OR) was prepared at a concentration of 10 mM in milli-Q water. Pyranine (final concentration of 300 *μ*M) was mixed with the stocked liposomes (4 mg of lipid) and 100 mM KPi (pH 7.0) in a total volume of 1 mL. Pyranine was encapsulated in the liposomes by three freeze-and-thaw cycles at 30°C (65°C for mixtures containing DPPE, DPPC, and DPPG lipids). The osmolality of the liposome lumen is ∼190 mosmol/kg. This value equals the osmolality of the assay buffer (100 mM KPi (pH 7.0)). After extrusion through a 200 nm polycarbonate filter at room temperature (65°C for mixtures containing DPPE, DPPC, and DPPG lipids) to homogenize the vesicles, the liposomes were eluted through a 22-cm-long Sephadex-G75 (Sigma-Aldrich) column pre-equilibrated with the assay buffer to remove the external pyranine. For blank correction, empty liposomes were prepared using the same procedure without the addition of pyranine. The collected 1 mL fractions containing the liposomes were identified using either an ultraviolet lamp (for liposomes filled with pyranine) or a NanoDrop spectrophotometer (for empty liposomes) and diluted in a total volume of 12 mL of the assay buffer.

### Stopped-flow experiments

A stopped-flow apparatus (SX20; Applied Photophysics, Leatherhead, Surrey, UK) operated in single-mixing mode was used to measure fluorescence intensity kinetics upon application of an osmotic shock to the liposomes filled with either calcein or pyranine. To impose the osmotic shock, the weak acid solution (∼300 mosmol/kg after mixing) and the liposome solution were loaded each in one syringe, and forced first through the mixer (1:1 mixing ratio and 2 ms dead time) and second into the optical cell (20 *μ*L volume and 2 mm pathlength). The temperature of the optical cell was set at 20°C using a water bath. The white light emitted by a xenon arc lamp (150 W) was passed through a high-precision monochromator and directed to the optical cell via an optical fiber. The band pass of the monochromator was optimized and set to 0.5 nm (for calcein) or 1.4 nm (for pyranine) to prevent fluoropore photobleaching during the experiment. The fluorophores were excited at 495 nm (for calcein) or at both 405 and 453 nm (for pyranine). The emitted light, collected at 90°, was filtered by a Schott long-pass filter (cutoff wavelength at 515 nm) and detected by a photomultiplier tube (R6095; Hamamatsu, Hamamatsu City, Japan) with 10 *μ*s time resolution. The voltage of the photomultiplier was automatically selected and kept constant during each set of experiments. The fluorescence intensity kinetics after the osmotic shock was recorded with logarithmically spaced time points to better resolve faster processes. For noise reduction, multiple acquisitions f_i_(t) (three for slow kinetics and nine for fast kinetics) were performed for each experimental condition.

### Preprocessing of the in vitro kinetic data

The raw data were preprocessed in MATLAB (R2015b; The MathWorks, Natick, MA) for further analysis. First, the N curves, which we called f_i_(t), acquired with a single experimental condition were averaged (F(t) = N^−1^∑f_i_(t)) to reduce the noise. For calcein, the resulting kinetic curves F(t) were normalized to 1 at time zero (F(t)/F(0)), i.e., the mixer dead time (t_0_ = 2 ms). For pyranine, the ratio r(t)_453/405_ was computed between the blank-subtracted kinetic curves collected at the two excitation wavelengths, i.e., F(t)_453_ and F(t)_405_. The pH(t) kinetic curves were calculated using the pyranine pH calibration curve (see next section).

### Pyranine pH calibration

A pH calibration curve was determined for the ratiometric fluorophore pyranine. Pyranine solutions (1 *μ*M) were prepared in 100 mM KPi in the pH range from 5.75 to 7.5 (±0.03 at ∼21.5°C). The fluorescence intensity upon excitation at both 405 and 453 nm was recorded for 30 s on the stopped-flow apparatus upon mixing with buffer. The ratio r = F_453_/F_405_ between the blank-corrected time-averaged intensities was calculated for each pH. The data points were fitted in MATLAB (curve fitting toolbox) with a biexponential empirical function: pH = a × exp (b × r) + c × exp(d × r), where a = 6.633, b = 0.1152, c = −1.009, and d = −9.241 (see Fig. S3). Later, the function was used to convert the measured ratio to pH values.

### Linear response of the calcein assay

The liposomes filled with calcein were tested for linearity between the fluorescence intensity drop *Δ*F = (F_unshocked_ − F_shocked_)/F(0) after osmotic upshift and the applied osmotic gradient (*Δ*Osm = Osm_out_ − Osm_in_). To this end, the DOPE:DOPG:DOPC liposomes were osmotically shocked with KCl at different concentrations (i.e., different osmolality) on the stopped-flow apparatus, and the fluorescence intensity kinetics was measured for each KCl concentration (see Fig. S4, *upper panel*). KCl was chosen because the K^+^ and Cl^−^ ions do not penetrate the lipid membrane on the timescale of the measurements. The intensity variation (*Δ*F) was plotted against the osmotic gradient (*Δ*Osm). Clearly, the plot (see Fig. S4, *lower panel*) is linear up to a gradient of ∼120 mosmol/kg. Thus, we can safely assume that with the applied experimental conditions (*Δ*Osm ∼110 mosmol/kg), the kinetic curves measured with the calcein self-quenching assay are devoid of nonlinearity effects.

### Fit of the in vitro kinetics

The function <F(t)>/<F(0)>, describing the time evolution of the calcein fluorescence, was calculated as described in the Appendix B of the accompanying work (20). In brief, the relaxation kinetics of the calcein concentration c_2_(r_0_,t) was computed by numerical solution of the system of differential equations describing the dynamics of a spherical vesicle of radius r_0_ upon osmotic upshift. The numerical solution was used to calculate the ratio F(r_0_,t)/F(0), using the Stern-Volmer equation with dynamic quenching constant K_SV_. The population-averaged ratio <F(t)>/<F(0)> was computed by using the vesicle size distribution g_i_(r_0_) measured in dynamic light scattering (DLS) experiments and fitted in MATLAB to the experimental data using the FMINUIT (22) minimization routine. For the “impermeable” osmolyte (KCl), two fitting parameters were used: the quenching constant K_SV_ (M^−1^) and the water permeability coefficient P_w_ (cm/s). For the permeable osmolytes (sodium pyruvate, lithium lactate, sodium formate, potassium acetate, and sodium benzoate), the water permeability coefficient P_w_ was fixed to the value obtained from the KCl data, whereas K_SV_ and P_AH_ were fitted to each other. To improve the accuracy and to estimate the error of P_AH_, we repeated the fit (at least 10 times) using different vesicle size distributions (see Fig. S5). The mean of the fitted values was used as the best estimate of P_AH_, and the standard deviation indicates the experimental uncertainty for the permeability coefficient *δ*P_AH_. The set of fitting parameters is presented in Tables S1 (for liposomes prepared from POPE:POPG:POPC lipids at a 2:1:1 weight ratio) and S2 (for liposomes prepared from lipids with a different degree of unsaturation of the acyl tails). The other parameters required for calculation of c_2_(r_0_,t) were set to their experimental values, which are pH_O_ = 7, [KPi]_I_ = 90 mM, [KPi]_O_ = 100 mM, c_2_(r_0_,0) = 10 mM, M_w_^H2O^ = 18 cm^3^/mol, pK_a_(KPi) = 7.21, and pK_a_(acid) = see Table 1. The subscripts I and O indicate the pH or concentration in the internal and external solution, respectively, and M_W_^H2O^ is the molar volume of water. The concentration of the weak acids [AH]_O_ in the external solution was set to ∼65 mM for all acids, with the exception of succinic acid, which was set to 47 mM, as obtained from Fig. S1. Accordingly, the total osmolyte concentration is 2[AH]_O_ to account for the counterion released by the weak acid salt. The complete set of parameters used for the analysis is given in the Appendix I.

**Table 1:** Molecular Weight And Pk_a_ Values Of The Used Osmolytes

Osmolyte	MW (g/mol)	pK_a_ (25°C)	Compound ID
KCl	74.55	N/A	–
Sodium succinate	162.05	4.21, 5.64	1110
Sodium pyruvate	110	2.45	1060
Lithium lactate	96.01	3.86	612
Sodium formate	68.01	3.75	284
Potassium acetate	98.15	4.76	176
Sodium benzoate	144.1	4.19	243

The pK_a_ values are found on the PubChem database (https://pubchem.ncbi.nlm.nih.gov/), using the compound ID indicated in the last column. N/A, not applicable.

### Determination of size distribution of liposomes

The size distribution of liposomes was measured by DLS using the DynaPro NanoStar Detector (Wyatt Technology, Santa Barbara, CA). Empty liposomes were prepared starting from 1 mg of lipids by three freeze-and-thaw cycles at 40°C. After 13× extrusion through a 200 nm filter, liposomes were eluted through a 22-cm-long Sephadex-G75 column pre-equilibrated with 100 mM KPi (pH 7.0). Before the DLS measurements, the liposomes were diluted with the assay buffer to a concentration in the range from 2 *μ*g/mL to 2 mg/mL. Measurements were performed with a scattering angle of 90°. For each measurement, at least 10 acquisitions of 20 s each were performed at a temperature of 20°C. For each acquisition, at least 2 million counts were recorded. The correlation curves and the intensity-weighted distributions were obtained with the built-in analysis software.

### Yeast strains and growth media

The *S. cerevisiae* strain IMK289 (23) was derived from CEN.PK102-3A (MATa *MAL1x MAL2x MAL3x leu2-112 ura3-52 MAL2-8*^*C*^) by replacement of the maltose metabolism loci *MAL1x*, *MAL2x*, *MAL3x*, *MPH2*, and *MPH3* with *loxP*. Subsequently, RA380 was derived from IMK289 by transformation with a plasmid (pYES2-Pact1-pHluorin with *ACT1* promoter and *URA3* selection marker) carrying the genetically encoded pH sensor pHluorin (24) and another plasmid (pRHA00L0 containing *LEU2*) to make the strain prototrophic (25). The MG10 strain was derived from the IMX1067 strain (MATa *ura3-52 trp1-289 leu2-3, 112 his3Δ1 MAL2-8c SUC2 can1::CAS9-natNT2 ITR1Δ PDR12Δ MCH1Δ MCH2Δ MCH5Δ AQY1Δ MCH3Δ MCH4Δ Yil166CΔ HXT1Δ JEN1Δ ADY2Δ AQR1Δ THI73Δ FPS1Δ AQY2Δ YII053cΔ ATO2Δ ATO3Δ YRO2Δ AZR1Δ TPO2Δ YHL008cΔ YFL054cΔ TPO3Δ* + pUDE412) (19) carrying the CEN.PK2-1C genetic background. The pUDE412 plasmid was cured by growth in yeast extract peptone dextrose (YPD) media to remove the selective pressure on the plasmid carrying the *URA3*, *LEU2*, *HIS3*, and *TRP1* markers. After 2 days, positive selection of cured cells was performed by streaking on SD + 5-FoA plates. Finally, the cells were transformed with the pYES2 plasmid (see above) carrying the genetically encoded pH sensor pHluorin. The Y7001 strain is derived from the IMX1000 strain (MATa *ura3-52 trp1-289 leu2-3*, *112 his3Δ1 MAL2-8c SUC2 can1::CAS9-natNT2 ITR1Δ PDR12Δ MCH1Δ MCH2Δ MCH5Δ AQY1Δ MCH3Δ MCH4Δ Yil166CΔ HXT1Δ JEN1Δ ADY2Δ AQR1Δ THI73Δ FPS1Δ AQY2Δ YII053cΔ ATO2Δ ATO3Δ YRO2Δ AZR1Δ TPO2Δ YHL008cΔ YFL054cΔ TPO3Δ* + pRSII425_Phluorin + pUDC013) (19) carrying the CEN.PK2-1C genetic background. Cells were transformed with the pRSII425-Phluorin plasmid, which is similar to pYES2 (see above) except that the *URA3* marker was replaced by *LEU2*; the cells were co-transformed with pUDC013 (pRS416-Ppgk1-ady2_L219V-Tcyc1) containing the lactic-acid-transporting L219V mutant variant of the *ADY2* gene with the *PGK1* promoter, *CYC1* terminator, and *URA3* marker. Synthetic complete drop-out (SD) medium lacking all amino acids (or uracil (URA) only for MG10 or uracil/leucine (URA/LEU) for Y7001) was made using 2% (w/v) glucose and yeast nitrogen base low-fluorescence without amino acids, riboflavin and folic acid (from Formedium; Norfolk, UK). Liquid cultures were inoculated in SD without amino acids (SD − URA for MG10 and SD − URA/LEU for Y7001) with a single colony from agar plates, and the cells were exponentially grown (optical density (OD) < 0.6) for at least 48 h at 30°C and with 200 rpm shaking. Before the experiment, to wash away the growth medium, the cells were pelleted by centrifugation (3000 rpm, 5 min, 4°C in an A-4-81 rotor of an Eppendorf 5810R centrifuge; Hamburg, Germany) and resuspended in 2 mL of 100 mM KPi (pH 6.0). The previous step was repeated, and the cells were resuspended to a final OD_600_ of ∼20 for measurements on the fluorometer or an OD_600_ of ∼2 for the stopped-flow experiments. The cell solution was kept on ice for the duration of the experiment. The OD_600_ values were measured with an Ultrospec 10 (Amersham Biosciences, Little Chalfont, UK) OD meter with 1 cm pathlength plastic cuvettes.

### Influx assay in vivo with fluorometer: Slow kinetics

The yeast cell suspension (OD_600_ ∼20) was first diluted to OD_600_ ∼2 in 100 mM KPi (pH 6.0) and then equilibrated at 30°C while recording the fluorescence emission of pHluorin. After 10 min, the solution was diluted to OD_600_ ∼0.1 in the 100 mM weak acid solution (pH 6.0) while still recording the pHluorin fluorescence. During the measurements, the solution was continuously stirred (600 rpm) with a magnetic bar. Fluorescence emission intensity was recorded with a JASCO FP-8300 fluorometer (JASCO, Tokyo, Japan) in dual-wavelength excitation mode. The solution was illuminated with monochromatic light at both 390 and 470 nm. The emitted light was collected at 512 nm with a right-angle configuration and a 1 cm pathlength. The monochromator bandwidths were set to 2.5 (excitation) and 5.0 (emission) nanometers, respectively. The data points were recorded every 6 s using an acquisition time of 100 ms. The pH was calculated from the fluorescence intensity ratio r = F_390_/F_470_, using the pHluorin calibration curve (see below).

### pHluorin pH calibration

Yeast solutions (2 mL) were prepared in 100 mM KPi in the pH range from 7.2 to 5.25 (±0.03 at ∼21.5°C) by diluting the cell suspension (OD_600_ ∼20) to OD_600_ ∼0.1 in the presence of 0.02% digitonin to permeabilize the plasma membrane. The digitonin 2% (w/v) stock solution was freshly prepared before the experiment by dissolving the powder at 95°C for 10 min in 100 mM KPi (pH 7.0). The cell solutions were incubated for 30 min at 30°C (600 rpm) to equilibrate the intracellular and the extracellular pH. The fluorescence emission was recorded in dual-wavelength excitation mode for 90 s as described above (see Influx Assay In Vivo With Fluorometer: Slow Kinetics)). The ratio r = F_390_/F_470_ between the time-averaged fluorescence intensities was calculated for each solution and plotted versus the pH value (see Fig. S7). The data points were fitted in MATLAB (curve fitting toolbox) with a biexponential empirical function: pH = a × exp(b × r) + c × exp(d × r), where a = 5.33, b = 0.1507, c = −5.195, and d = −5.109.

### Influx assay in vivo with stopped flow: Fast kinetics

To resolve fast pH kinetics in vivo (sodium benzoate and potassium acetate), we performed the pHluorine pH assay on the stopped-flow apparatus. The yeast suspension (OD_600_ ∼2) and the 100 mM weak acid solution (sodium benzoate or potassium acetate), both in 100 mM KPi (pH 6.0), were each loaded in one syringe and pre-equilibrated at 30°C for at least 5 min. Importantly, before each recording, the cell solution was mixed inside the syringe to dissipate concentration gradients. Furthermore, five mixing cycles were required to obtain a homogeneous concentration inside the optical cell and get a steady signal. Accordingly, for potassium acetate, five mixing cycles were always performed before and after a 5 min recording. This procedure was repeated until a total number of three recordings were obtained, whereas for sodium benzoate, six consecutive recordings of 10 s were performed after the five mixing cycles. Fluorescence kinetics was recorded following the same procedure as described for the pyranine assay (see above, Stopped-Flow Experiments) with the exception of 1) the excitation wavelength (390 and 470 nm instead of 405 and 453 nm) and 2) the bandwidth of 4.65 nm instead of 1.4 nm.

### Fit of the in vivo kinetics

To fit the in vivo pH kinetics data with the theoretical model presented in the accompanying work (20), we numerically solved the system of differential equations describing the yeast cell dynamics upon osmotic upshift with a weak acid. From the proton concentration in the yeast cytosol, we calculated the time evolution of the internal pH, that is, pH_I_(t). To obtain the permeability coefficient of weak acids across the yeast plasma membrane, we fitted the relaxation curves by minimization of the sum of squared residuals with FMINUIT (22) in MATLAB. The fitting parameters are the weak acid permeability coefficient and an effective KPi concentration; the latter reflects the overall buffering capacity of phosphates in the cell (free inorganic phosphate, protein-bound phosphate groups, organic phosphates, polyphosphate); the complete set of parameters is given in Appendix I.

We choose the parameters as follows: we consider a spherical cell with volume V_0_ = 81.9 fL, a volume V_r_ at zero-turgor pressure of 66.6 fL (also called zero-turgor volume), and nonosmotic volume b = 0.65V_r_ = 43.3 fL (26). During the experiment, the yeast solution was kept at 30°C (303 K), in contact with air at atmospheric pressure (∼101.3 kPa), and well mixed at 600 rpm. We assume that before the osmotic upshift, at time t < 0, the cell is in a stationary state. The osmotic volume V_0_ − b = 38.6 fL is filled with an aqueous solution at pH ∼6.5 (see Fig. 6) containing solute molecules and the pH probe pHluorin (6). The most abundant solutes are ions (K^+^, Na^+^, Mg^2+^, SO_4_^2−^, PO_4_^3−^) and free amino acids (mostly glutamate) with a total concentration of ∼405 mM in 38.6 fL (27). The internal solution is buffered by carbon dioxide (CO_2_(aq)), which at pH 6.5 dissociates according to the following equilibrium:

$$CO_{2}\left(aq\right)⇋HCO_{3}^{-}+H^{+}.$$

The effective KPi concentration accounts for the total concentration of the phosphate groups (∼230 mM in 38.6 fL) present in the cytosol (data from (27)). The phosphates are either bound to other molecules (phosphorylated proteins and polyphosphates) or free in solution as inorganic and organic phosphate (27). Importantly, the aforementioned concentrations were corrected to account for the dilution factor 38.6 fL/30 fL = 1.29 of the nonosmotic volume V_0_ − b with respect to the reference volume of 30 fL given in (27). A CO_2_(aq) concentration of ∼11.3 *μ*M was estimated at atmospheric pressure and 303 K from the Henry solubility constant, H_cp_(303 K) = 2.8 × 10^−4^ mol/(m^3^ × Pa) (28), using a molar fraction of CO_2_ (g) in air of 4 × 10^−4^. Because CO_2_(aq) is highly permeable across lipid membranes (29), i.e., on the timescales (>10 s) of our experiments, the CO_2_(aq) internal concentration is equal to the external concentration and independent from the cellular volume at each time. The relevant pKa value of CO_2_(aq) is 6.73. We set the pKa of KPi to the pKa of the yeast cytosol as measured in (30). The external solution, at pH 6, contains 100 mM KPi and 11.3 *μ*M of CO_2_(aq).

At time t = 0, we osmotically upshift the vesicle solution by mixing it with 100 mM KPi (pH 6) containing 100 mM of a weak acid salt (potassium acetate or sodium formate). Under these conditions, the external osmotic pressure Π_e_ ∼ RTc_s_^∗^ varies between 0.25 and 0.75 MPa. Thus, the cellular volume V is always larger than the zero-turgor volume, that is, V > V_r_ (see (26), in which the ratio V/V_r_ was measured as a function of *Π*_e_). Also, a ratio between the major/minor axes of 1.14 was measured for yeast cells with an external osmotic pressure of 1.4 MPa (26), showing that the spherical approximation is reasonable at 0.25 MPa. The volumetric elastic modulus is set to *ϵ* = 4.53 MPa. The values of *ϵ* is obtained from a linear fit of the data reported by Smith et al. (see Fig. 11 in (31)) with the following equation (data not shown):

$$\Delta P_{M}=\varepsilon \frac{\Delta V}{V_{r}}.$$

After the osmotic upshift, the cell relaxes to equilibrium by exchanging mass and volume with the external solution. Among the molecules considered in our description, only three have permeability coefficients sufficiently high to diffuse passively across the membrane on relevant timescales (1 s to 0.5 h). These molecules are water, CO_2_, and the weak acid AH. The permeability of water in yeast P_w_ = (10^−1^–10^−2^) cm/s is unexpectedly high, and the microscopic mechanism determining such a behavior is still under debate (31,32). The permeability of carbon dioxide has, to the best of our knowledge, not been measured in yeast. Thus, we set CO_2_ permeability to the lowest value of 10^−3^ cm/s reported in the literature for the apical epithelial membrane of the guinea pig colon (29). The permeability of weak acids across the yeast plasma membrane is unknown.

### Estimate of unstirred layer contribution

To estimate a possible contribution of unstirred layers to the measured permeability ratio P_vesicle_/P_cell_, we consider the relationship between the observed “apparent” permeability P and the actual membrane permeability P′ (33,34)

$$P=\frac{P'}{1+P'\frac{\delta }{D}}.$$

where *δ* (*μ*m) is the thickness of the unstirred layer and D (cm^2^/s) is the diffusion coefficient of the permeant in solution. Upon setting P_vesicle_ = P_v_ and P_cell_ = P_c_, we write the ratio

$$\frac{P_{v}}{P_{c}}=\frac{P{'}_{v}}{P{'}_{c}}\times \frac{D_{v}}{D_{c}}\times \frac{D_{c}+\delta_{c}P{'}_{c}}{D_{v}+\delta_{v}P{'}_{v}}.$$

Then, we exploit the proportionality between the maximal thickness of the unstirred layer *δ* and the characteristic length *l* of the object considered (34) (for a spherical object like a vesicle or a cell, *l* corresponds to the diameter), that is, $\delta \;\propto \sqrt{l}$. We then obtain

$$\delta_{v}=\delta_{c}\frac{\sqrt{l_{v}}}{\sqrt{l_{c}}}.$$

Finally, we substitute *δ*_*v*_ in the previous equation to obtain

$$\frac{P_{v}}{P_{c}}=\frac{P{'}_{v}}{P{'}_{c}}\times \frac{D_{v}}{D_{c}}\times \frac{D_{c}+\delta_{c}P{'}_{c}}{D_{v}+\delta_{c}\sqrt{\frac{l_{v}}{l_{c}}}P{'}_{v}}.$$

We use this equation to estimate the maximal possible contribution of unstirred layers to the ratio P_v_/P_c_. To this end, we assume that the actual permeability in vesicles and in cells is the same, that is, P′_v_ = P′_c_. Also, we set the diffusion coefficient in the aqueous solution D_*v*_ to 10^−5^ cm^2^/s (35) and the characteristic lengths *l* to 5.4 *μ*m (cell) and 0.25 *μ*m (vesicle). We set the diffusion coefficient inside the cell to D_*c*_ = D_v_/8. To our knowledge, the diffusion coefficient of small molecules in the yeast cytosol was never measured. However, the diffusion coefficients of small solutes and small proteins were measured in *Escherichia coli* (36), fibroblasts (37), *Xenopus* oocytes (38), and *Dictyostelium* (39), showing a 2- to 8-fold difference with respect to the value in aqueous solution. The thickness of the unstirred layer of the cell was varied from 0.001 to 5.4 *μ*m because the width of the unstirred layer cannot exceed the size of the object (here the vesicle or cell). The computed curves are plotted in Fig. S8, showing that unstirred layers account at maximum for a 35-fold difference between the ratio of vesicle/cell permeability. The estimated ratio P_v_/P_c_ is most likely overestimated because simulations of diffusion coefficients of small molecules in crowded solutions approach the values in aqueous solution (40,41). Hence, a more realistic estimate of P_v_/P_c_ is fivefold.

## Results and Discussion

### Design of the in vitro experiments

To monitor the permeability of weak acids (benzoic, acetic, formic, lactic, pyruvic, and succinic acid) and water through the liposome membrane, two independent and complementary fluorescence-based kinetic assays were designed. The first assay reports volume changes of liposomes by means of calcein self-quenching fluorescence (42). The second assay detects the pH variation in the liposome lumen using the ratiometric fluorophore pyranine (43). Both assays exploit the out-of-equilibrium relaxation kinetics of liposomes after the increase of the external osmotic pressure (osmotic upshift), i.e., the addition of an osmolyte to the liposome solution. Indeed, after the osmotic upshift, the thermodynamic equilibrium can be re-established by the flux of 1) water (to re-equilibrate the chemical potential of water) and/or 2) the osmolyte (to dissipate the osmolyte concentration gradient) (33). The contribution of the two fluxes to the recovery kinetics depends on the relative permeability of water and the osmolyte. Thus, the two independent kinetic assays allow for determination of permeability coefficients of both water and the osmolyte.

### Internal volume measurements, qualitative description

**Figure 1:**
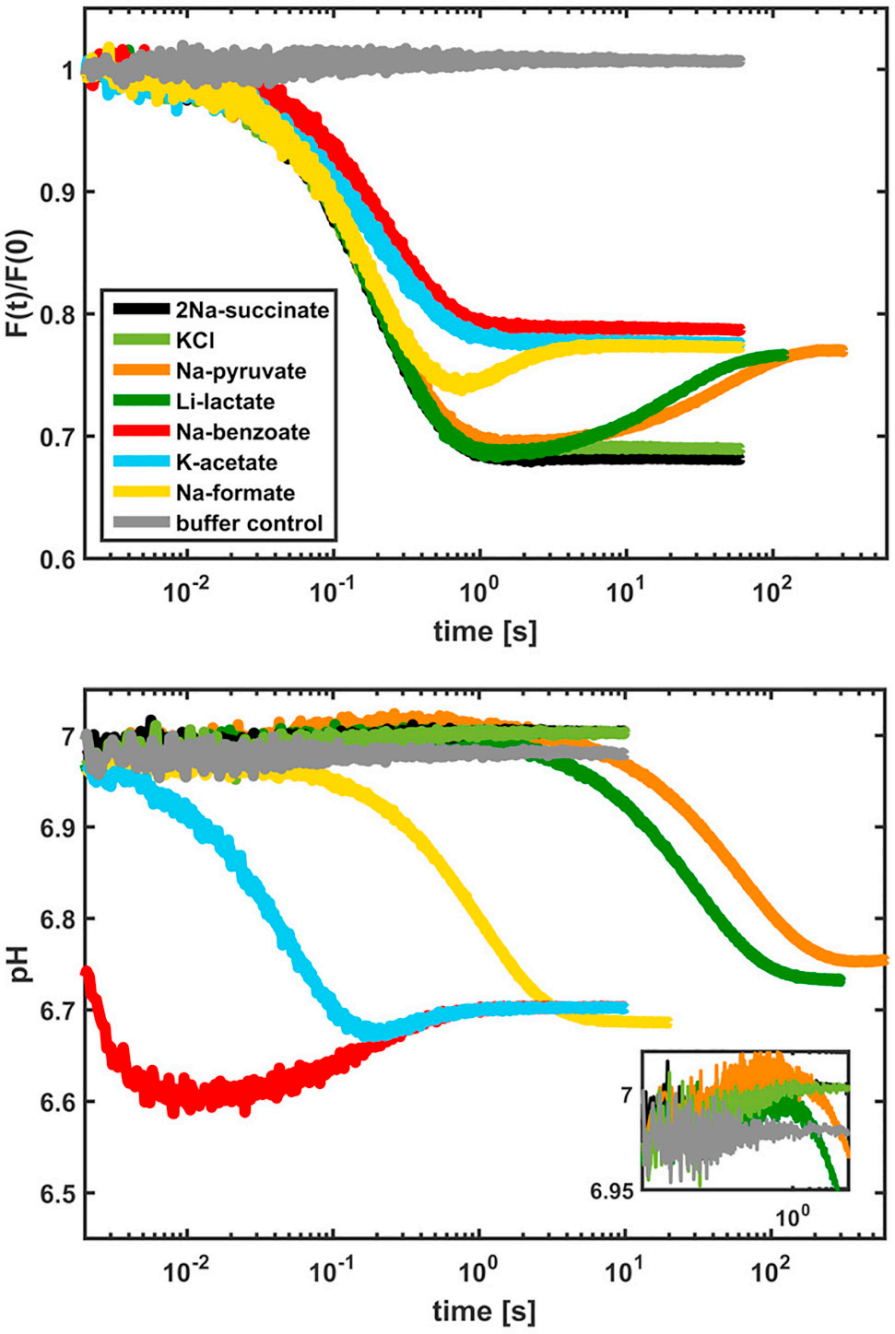
Kinetic data obtained with the calcein self-quenching assay (*upper panel*) and the pyranine pH assay (*lower panel*) using liposomes composed of POPE:POPG:POPC at a 2:1:1 weight ratio. The color code is the same for both panels. To see this figure in color, go online.

In the calcein (de)quenching assay, liposomes filled with calcein at a self-quenching concentration were used (42). Here, changes of the fluorescence intensity reflect variations of the liposome internal volume, which are caused by fluxes of water and osmolytes through the membrane; when the volume decreases, the intensity also decreases, and vice versa. The fluorescence intensity kinetics normalized to time zero for the POPE:POPG:POPC (2:1:1 weight ratio) lipid mixture is shown in Fig. 1 (*upper panel*); the properties of the osmolytes tested are given in Table 1.

The data give insight into the microscopic behavior of the lipid vesicles. In brief, we show that after osmotic perturbation, weak acids (protonated state), weak bases (deprotonated), and water move across the membrane to establish partial (counterion is nonpermeable) or full diffusion equilibrium (all species permeable on the timescale of the measurements). Specifically, after the osmotic upshift (in the time range from 2 to 300 ms), the fluorescence intensity decreases immediately, indicating liposome shrinkage. Once the system has reached the kinetic equilibrium, two states are observed: 1) a fully shocked state (F(t)/F(0) ∼0.7) and 2) a partially recovered state (F(t)/F(0) ∼0.8). Knowing that on the timescale used in this experiment (≤300 s), the membrane is impermeable to KCl (the permeability of K^+^ and Cl^−^ is ∼10^−12^ and ∼10^−9^ cm/s, respectively (44,45)) but permeable to water (∼10^−2^ cm/s (44)), we attribute the fast kinetic process (<300 ms) to water efflux and the F(t)/F(0) ∼0.7 state to maximally shrunk liposomes (Fig. 1, *light green curve*). By similarity with KCl, sodium succinate is also membrane impermeable (Fig. 1, *black line*). At intermediate timescales (from ∼300 ms to 20–300 s), different interconversion rates between the two states are observed for three of the tested salts: sodium formate (*yellow*) > lithium lactate (*dark green*) > sodium pyruvate (*orange*). We assign this behavior to the weak acid (osmolyte) diffusing across the membrane and partially recovering the internal volume.

The recovery is partial because the counterions (Na^+^ or K^+^) are expected to be membrane impermeable on this timescale (see above for K^+^ and (46) for Na^+^ having a permeability of ∼10^−14^ cm/s).

**Figure 2:**
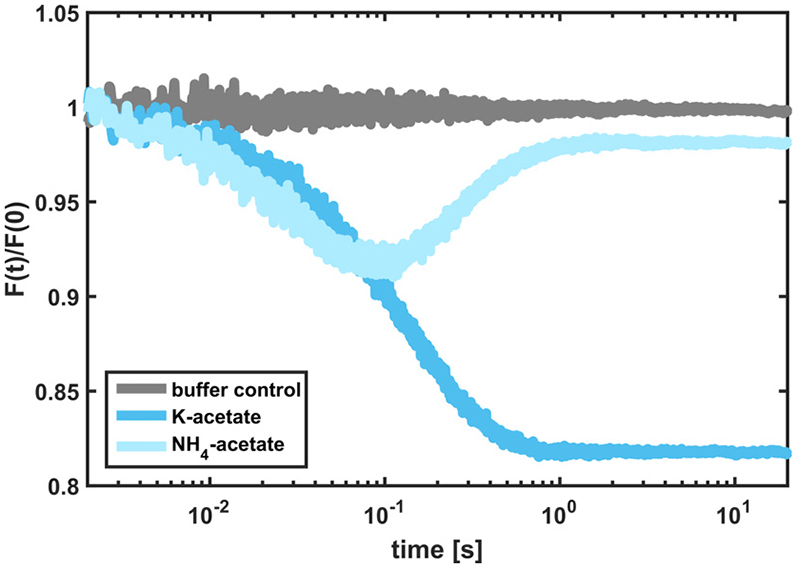
Kinetic data obtained with the calcein self-quenching assay using liposomes composed of POPE:POPG:POPC at a 2:1:1 weight ratio. To see this figure in color, go online.

To test this hypothesis, we performed a calcein kinetic experiment using ammonium-acetate salt (NH_4_-acetate), which in solution generates two membrane-permeable species: 1) ammonia with a permeability coefficient of ∼10^−1^ cm/s (47) and 2) acetic acid. In Fig. 2, we compare the kinetic data to the curve obtained with potassium acetate. Clearly, the signal of NH_4_-acetate (*light blue*) almost returns to the starting value within ∼2 s, which points to recovery of the liposome volume and of the osmotic equilibrium (Osm_O_ − Osm_I_ = 0 for t > 2 s). To strengthen our interpretation, we conducted pH kinetic measurements with NH_4_-acetate (Fig. S9). Here, alkalinization of the vesicles is followed by acidification, confirming the influx of ammonia and acetic acid, respectively. Thus, we conclude that both the ammonia and the acetic acid molecule diffuse inside the vesicles and restore the osmotic balance on the timescale of our experiments. Finally, we observe in Fig. 1 (*upper panel*, *red* and *blue lines*) that two of the tested salts (sodium benzoate and potassium acetate) directly level off to the partially recovered state, indicating that the permeability of the acid component of the osmolyte pairs is comparable to (or higher than) that of water.

### Internal pH measurements (qualitative description)

Next, we determined which of the osmolyte species, the anion (A^−^) or the acid (AH) form, has the higher permeability. Based on energetic considerations and experimental data, we expect a charged molecule to be at least four orders of magnitude less permeable than the neutral counterpart (44, 45, 46, 47, 48, 49, 50). Thus, we assume that the acids in their neutral form (AH) penetrate the vesicles, whereas the lipid membrane is impermeable to the anion (A^−^). Consequently, the inside of the vesicles should acidify because of a net flux of protons, carried by the permeant species (AH) into the vesicles, which have a limited buffering capacity and finite volume. To verify this hypothesis, a pH kinetic assay was performed using liposomes filled with the ratiometric pH-sensitive fluorophore pyranine (43) upon addition of the weak acid salts to the solution. In this assay, the ratio between the fluorescence intensities, recorded upon excitation at both 405 and 453 nm, monitors the pH inside the liposomes (see Materials and Methods). The results are shown for the POPE:POPG:POPC (2:1:1 weight ratio) lipid mixture in Fig. 1 (*lower panel*). Strikingly, all salts except sodium succinate induced a pH drop from 7.0 to around 6.7. Furthermore, the relative permeability of the weak acids is clearly distinguishable: benzoic > acetic > formic > lactic > pyruvic > succinic. Thus, the pH assay confirms the hypothesis that on the observed timescales, the liposomal membrane is only permeable for the acidic form (AH) of the osmolyte.

More features are distinguishable from the data. Both KCl and sodium succinate pH kinetics are fairly constant in time and behave similarly to the buffer control. However, a small increase of ∼0.02 pH units is observed at around 100 ms (see *inset* of Fig. 1, *bottom panel*) with respect to the control experiment. A similar increase is seen for potassium acetate, lithium lactate, and sodium pyruvate. Because liposomes shrink on the same timescale as water effluxes, as shown by the calcein assay (see *upper panel*), we attribute the small increase in pH to an increment of the ionic strength due to the increasing KPi concentration inside the liposomes. To confirm the hypothesis, we measured the fluorophore readout at pH 7.0 in KPi buffer solutions at concentrations from 100 to 500 mM (see Fig. S10). Clearly, an apparent increase of pH is observed at higher buffer concentration, that is, at higher ionic strength. Sodium benzoate also shows an increase in pH at around 100 ms. However, the pH variation is up to two orders of magnitude larger with respect to the other acids (almost 0.1 pH unit, compared to 0.02). Thus, the ionic strength dependence alone cannot explain such a large pH increase. To account for the extra contribution, we notice that the acid influx precedes the water efflux. Therefore, the subsequent water efflux, which causes shrinkage of the liposomes, raises the internal acid concentration above the value in solution (AH_IN_ > AH_OUT_). To dissipate the acid concentration gradient, a reflux of the acid takes place leading to the observed pH increase inside the liposomes. Finally, to rule out the effect of an open buffer like CO_2_, we compared the pH kinetics measured in solutions saturated with CO_2_ or N_2_ (see Fig. S11). Clearly, the difference between the two kinetic curves is minimal, showing that the contribution of the open buffer is insignificant for the analysis of the data.

### Physiochemical model of vesicle dynamics, quantitative description

**Figure 3:**
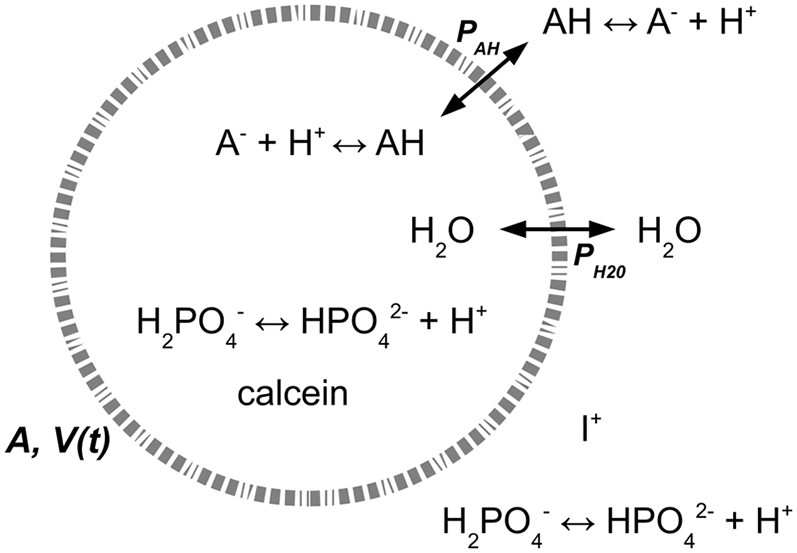
Schematic representation of the acid-base equilibria inside and outside the vesicles and the fluxes of weak acids and water across the membrane. *P*_*AH*_ and *P*_*H2O*_ refer to the weak acid and water permeability, respectively; *A* is surface area of the vesicle; *V*(*t*) is the volume of the vesicle.

To obtain a quantitative description of the data, we built a physiochemical model describing the vesicle relaxation dynamics upon osmotic upshift with a weak acid (salt) (20). The model encompasses the relevant features observed in the kinetic experiments (see Fig. 3), which are 1) permeation of water and weak acid across the membrane, 2) variation of the vesicle volume, 3) buffer capacity, 4) protonation and deprotonation of the weak acids, 5) heterogeneity of the vesicle size, and 6) calcein self-quenching. The model assumes 1) a vesicle with fixed surface area, 2) a membrane thickness much smaller than the vesicle radius, 3) well-mixed and dilute solutions of weak acids and buffer, 4) electrically neutral solutions, 5) a nanoscopically homogeneous membrane composition, and 6) a freely deformable membrane. Finally, we assume that the external solution is an infinite source of molecules. A detailed description of the model is given in the accompanying study (20). To obtain the permeability coefficients of water and the weak acids, we solved the equations describing the relaxation dynamics of the vesicles. Then, we fitted the time evolution of the calcein fluorescence, that is, F(t)/F(0), to the kinetic curves (see Materials and Methods, Fit of the In Vitro Kinetics).

**Figure 4:**
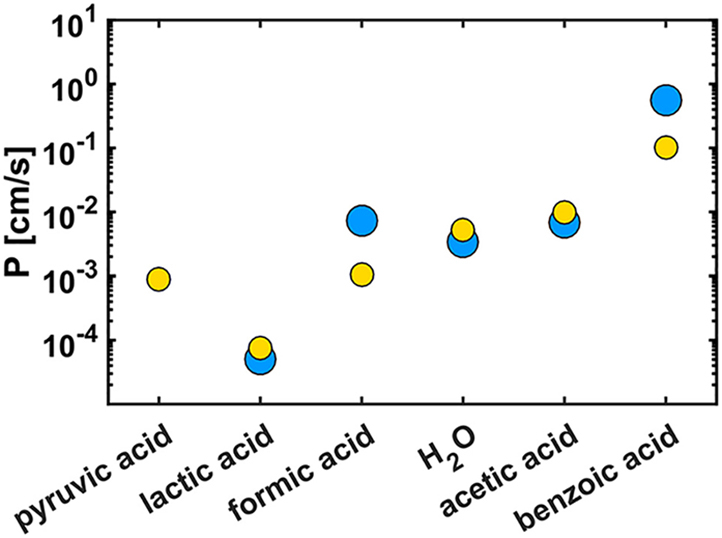
Permeability coefficients (cm/s) (*yellow circles*) of water and the weak acids in liposomes prepared from the POPE:POPG:POPC lipid mixture (2:1:1 weight ratio). *Blue circles*: permeability (cm/s) measured by Walter et al. using egg phosphatidylcholine lipid membranes (47). To see this figure in color, go online.

The fitting parameters and the fitted data of the various salts/weak acids are shown in Table S1 and Fig. S12, respectively. The permeability coefficients P (cm/s) (*yellow circles*) are plotted in Fig. 4 for the tested weak acids and vesicles composed of the POPE:POPG:POPC lipid mixture. Here, we point out that the values obtained for benzoic acid are not very accurate because of the very fast kinetics (see Fig. 1, *lower panel*). We observe that the permeability coefficients are in good agreement with the values measured by Walter et al. (*blue circles*) using egg phosphatidylcholine lipid membranes (47). Therefore, we conclude that the assay is perfectly suited to perform reliable permeability measurements in liposomes using not only weak acids but also weak bases (like ammonia) and other small molecules (like glycerol) or a combination of these.

### Membrane permeability as a function of lipid tail saturation

To better explore the potential of our permeability assay, we performed experiments on liposomes prepared from lipids with varying degrees of saturation of the lipid acyl chains and keeping the composition of the polar heads (2:1:1 weight ratio of PE:PG:PC = XX) constant. The degree of unsaturation defines the ratio between the number of lipid tails with carbon-to-carbon double bonds (N_C=C_) and the total number of tails (N): d = N_C=C_/N. Here, it is important to remember that the lipids have two tails per head and maximally one double bond per tail. In our experiment, we characterized six different lipid mixtures having degrees of unsaturation of 1, 0.84, 0.67, 0.5, 0.34, and 0.17 and tested two weak acid salts, lithium lactate and sodium formate. Pure mixtures of DOXX and POXX were prepared having degrees of unsaturation of 1 and 0.5, respectively. The four intermediate d-values were obtained by mixing DOXX and POXX or POXX and DPXX (see Materials and Methods); DPXX lipids have only saturated acyl chains (d = 0).

**Figure 5:**
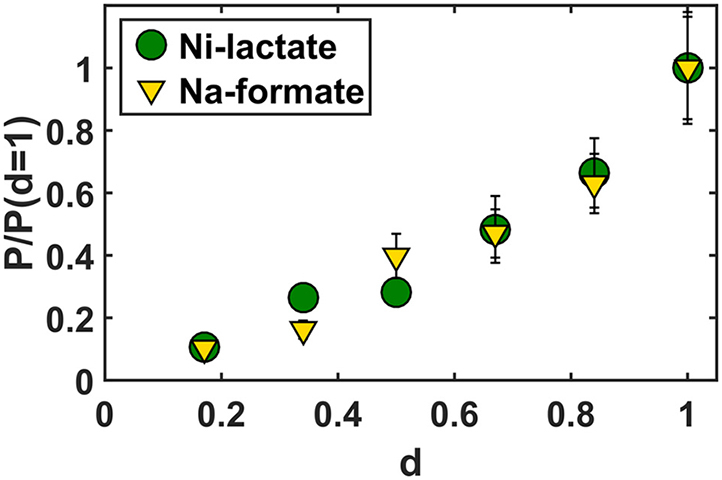
Permeability coefficients P (cm/s) (*green circles* and *yellow triangles*) of lactic and formic acid in liposomes prepared from lipid mixtures differing in degrees of acyl chain saturation (d); d is 1, 0.84, 0.67, 0.5, 0.34, and 0.17. The error bars displays the experimental error of the measured permeability coefficients. *p*-Values are normalized to the permeability coefficients at d = 1; P_formic_ = 7.4 × 10^−3^ cm/s and P_lactic_ = 0.12 × 10^−3^ cm/s at d = 1. To see this figure in color, go online.

To obtain the permeability coefficients of water and the weak acids, we fitted the calcein relaxation curves of the six mixtures (see Table S2). In Fig. 5, we display the normalized permeability coefficients (*green circles* and *yellow triangles*) of lactic acid and formic acid. The error bars indicate the experimental errors of the measured permeability coefficients. These errors originate mainly from the inaccuracy of the vesicle size distribution (see Materials and Methods). Strikingly, the data collapse on the same curve, showing that variations of the membrane permeability are independent of the chemical nature of the permeant. We conclude that the permeability assay allows the characterization of membrane permeability as a function of the lipid composition (here, the acyl chain saturation).

### Membrane permeability of yeast cells

**Figure 6:**
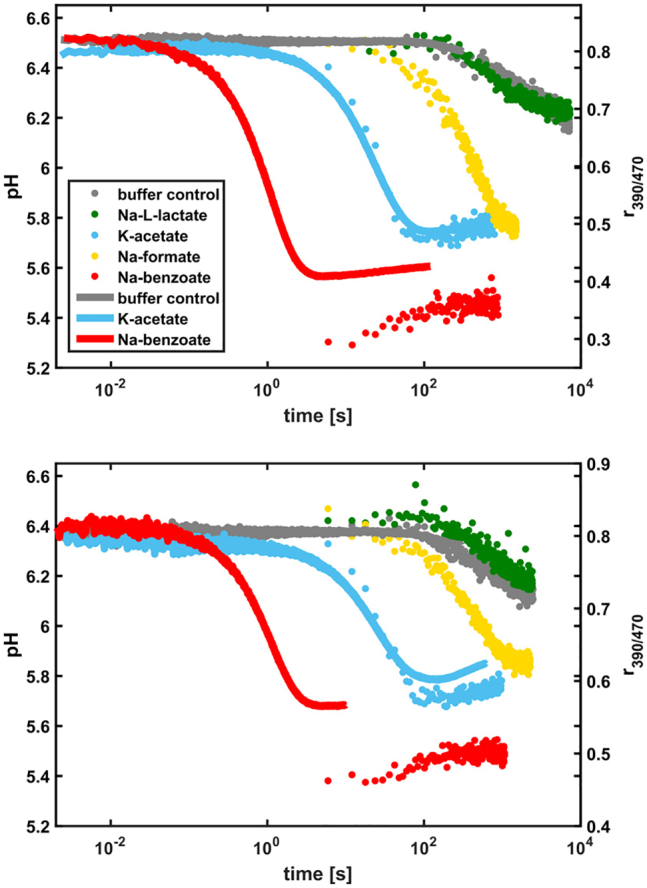
pH kinetics measured in vivo with pHluorin expressed in the *S. cerevisiae* strains RA380 (*upper panel*) and MG10 (*lower panel*). The color code is the same for both panels. The fast kinetics (*continuous line*) was resolved in the stopped-flow measurements and the slow kinetics (*dots*) with the fluorometer. The stopped-flow kinetics is plotted as the fluorescence intensity ratio r_390/470_ instead of pH. To see this figure in color, go online.

To determine the permeability of the yeast plasma membrane for weak acids, we performed an in vivo assay conceptually similar to the pyranine-based pH assay. We expressed the gene encoding a pH-sensitive fluorescent protein called pHluorin (6) in three *S. cerevisiae* strains: 1) a reference strain with all known acid transporters (RA380); 2) a strain (MG10) with deletion of 25 genes encoding all known and putative carboxylic acid transport proteins, including the complete aqua(glycero)porin family (19); and 3) a strain (Y7001) containing the 25 gene deletions but expressing the engineered acetate/lactate/pyruvate importer Ady2 L219V (11). We measured the changes in pH upon addition of weak acid salts to the cell suspension as a function of time. We compared the pH kinetics of the three strains to disentangle passive diffusion from active transport and/or facilitated diffusion. Indeed, we expect that transporters and facilitators, if expressed, would contribute an extra kinetic term with respect to the deletion strain. To resolve both slow and fast kinetics, the pH kinetics was recorded with a fluorometer, the stopped-flow apparatus, or both. Qualitatively, the pH kinetics (Fig. 6) show a pH drop followed by a slower pH recovery (see *red* and *blue traces*). Clearly, the pH drop tells us that a molecular species carrying a proton across the membrane and releasing it on the inside enters the intracellular milieu, most likely the weak acid in its neutral form (AH). We assign the slow recovery to the activation of the plasma membrane ATPase H^+^ pump (Pma1) that consumes ATP to pump out H^+^, thereby partially restoring the intracellular pH. Furthermore, the control experiment (in gray) shows a pH decrease after ∼100 s that we attribute to limited availability of ATP in the cell; the cells were suspended in buffer at pH 6 without a carbon source, which implies that little additional ATP is produced in the course of the experiment. Remarkably, we observe that the permeability of the weak acids follows the same order observed in vitro: benzoic > acetic > formic > L-lactic (see Fig. 1, *lower panel*). Here, the L-lactic acid permeation is not detectable on timescales up to 2 h even if the pH of the KPi solution is lowered to 5.2 and the sodium-L-lactate concentration is increased to 200 mM (see Fig. S13) to increase the concentration gradient of the permeant species (AH).

To obtain values of the permeability coefficient across the yeast plasma membrane, we fitted the relaxation pH kinetics measured on the fluorometer for potassium acetate and sodium formate by using a modified version of the mathematical model described above (see Fig. S14; Materials and Methods, Fit of the In Vivo Kinetics; and Appendix B of the accompanying work (20)). With respect to the lipid vesicles, the model for a yeast cell comprises 1) the volume, occupied by organelles and macromolecules, that is inaccessible to the solute molecules; 2) the contribution of free ions and amino acids to the internal osmotic pressure; 3) the elastic properties of the cell wall, which generates turgor; 4) the passive and channel-mediated permeation of water molecules through the plasma membrane; and 5) the buffering capacity of an open buffer (like CO_2_). The fitted permeability coefficients are reported in Table 2. We observe that the permeability coefficient for acetic acid (1.1 × 10^−5^ cm/s) is almost identical to that of formic acid (1.4 × 10^−5^ cm/s). Strikingly, the permeability coefficients across the yeast plasma membrane are 100 (formic acid)- to 750 (acetic acid)-fold smaller than in the POXX lipid vesicles (see Table 2). We estimate that unstirred layer effects may account for at most a fivefold difference in the ratio of the in vitro/in vivo permeability (see Materials and Methods, Estimate of Unstirred Layer Contribution and Fig. S8). The low permeability of the yeast plasma membrane is even more evident for lactic acid, which does not permeate over the 2 h timescale of our measurements. Thus, the very low permeability coefficients measured in vivo are an intrinsic property of the yeast plasma membrane, which warrants future investigation of the molecular basis for this difference.

**Table 2:** Permeability Coefficients in cm/s of Weak Acids Obtained from the In Vitro Vesicle and In Vivo Yeast Data

Weak Acid	P_*vesicles*_ (10^−^^5^) (cm/s)	P_*yeast*_ (10^−^^5^) (cm/s)	P_*vesicles*_/P_*yeast*_
Pyruvic acid	90	N/A	N/A
Lactic acid	6	N/A	N/A
Formic acid	210	1.1 ± 0.1	192
Acetic acid	990	1.4 ± 0.2	707
Benzoic acid	10,000	N/A	N/A

For the in vivo data, we report the mean value and standard deviation of the two strains (MG10 and RA380). The full set of fitting parameters is shown in Tables S1 and S3. N/A, not applicable.

The minor difference between the permeability coefficients of the RA380 (reference strain) and MG10 (25-fold knockout) (Table S3) indicates that weak acid symporters, if expressed in the reference strain, are not contributing much to the permeability under our experimental conditions as previously reported (18), thus suggesting that we are (mostly) probing passive diffusion of weak acids through the yeast plasma membrane rather than protein-mediated transport. To substantiate this finding, we compared the kinetics of weak acid diffusion in *S. cerevisiae* MG10 to that of Y7001 (Fig. S15); the Y7001 strain expresses the acetate/lactate/pyruvate importer Ady2 L219V (11) from the glucose-inducible *PGK1* promoter. Clearly, the kinetics of weak acid diffusion across the plasma membrane of the Y7001 strain do not differ from MG10, confirming that the major contribution to the observed pH drop is due to passive diffusion.

Finally, we comment on the estimated phosphate concentration (160–320 mM; see Table S3), which is obtained from fitting of the pH kinetics in vivo. Although this value is larger than the estimated 41 mM intrinsic buffering capacity of a yeast cytosol extract (30), our value is in good agreement with the total phosphorus concentration of ∼300 mM in yeast (free + bound phosphate groups, organic phosphates, polyphosphate) (27). We assume that phosphate present in metabolic intermediates (e.g., sugar phosphates) and bound to proteins contributes to the overall buffering, similar to inorganic phosphate; the pKa value of phosphate in sugar phosphates is similar to that of inorganic phosphate.

## Conclusions

In this work, we present a (stopped-flow) fluorescence-based assay for quantitative determination of permeability coefficients of weak acids (small molecules) both in lipid vesicles and in living cells. In vitro, the assay has proven able to measure the membrane permeability of weak acids and water. We believe that the method can serve other purposes, such as 1) to correlate protein-mediated transport activity to the membrane physical properties; 2) to relate membrane physical properties to lipid composition and temperature; and 3) to measure the permeability of membranes for molecules like glycerol, sugars, and other metabolites. The increased permeability of protocellular membranes for particular molecules (ribose, for instance) is an example of a possible evolutionary mechanism for a biochemical pathway (51, 52, 53) that can now be tested in greater depth. In the in vivo domain, there are numerous examples of organisms that differ in their sensitivity toward weak acids, alcohols, and drugs, and given species pose a health and safety problem, but the molecular basis (membrane lipid composition, passive diffusion, efflux pumps) for the differences is, in most cases, not known; hence, the need for a robust method to determine the permeation of molecules through synthetic and biological membranes.

We also determined the relation between the membrane permeability and the degree of acyl chain saturation of lipid mixtures. In vivo, the assay allows one to discriminate passive diffusion of weak acids from active transport and facilitated diffusion, provided suitable mutants and/or expression conditions are available. We determined the permeability of the yeast plasma membrane for weak acids. Importantly, we find that the yeast plasma membrane is highly impermeable to lactic acid when compared to that of, e.g., bacteria ((54); unpublished data), relatively impermeable to acetic and formic acid, and highly permeable to benzoic acid.

The comparison of in vitro with in vivo results permits better exploration of the microscopic origin of biological membrane properties. For the tested weak acids, we observed the same order of permeability in vitro and in vivo (benzoic > acetic > formic > lactic). However, the absolute values differ enormously, with the permeability coefficients for the yeast plasma membrane being much slower than those of vesicles prepared from POPE:POPG:POPC. We note that the order of the permeability coefficients is in agreement with the degree of toxicity of the acids in bacteria that have been investigated (8,34,55,56). The extremely low permeability of the yeast plasma membrane (most evident for lactic acid) parallels the very slow lateral diffusion of membrane proteins, which are three orders of magnitude slower than in bacterial or mammalian plasma membranes (57). We emphasize that we find no measurable permeation of lactic acid across the yeast plasma membrane over a period of ∼2 h, whereas the permeation occurs on the timescale of seconds in the vesicles. Thus, irrespective of how the data analysis is done to obtain permeability coefficients, these measurements show that the yeast plasma membrane is highly impermeable to lactic acid. The low membrane permeability allows yeast to maintain large concentrations gradients of molecules (e.g., glycerol) that otherwise would leak in or out, as is the case in, e.g., *E. coli*. Further studies are required to explain the molecular basis for the low permeability of the yeast plasma membrane for small molecules. The trend shown in Fig. 5 indicates that the degree of saturation of the acyl chains is an important factor. A degree of saturation of ∼0.58 (58), which was measured in yeast grown aerobically with glucose as a carbon source, would lead to a permeability comparable to that of POXX lipid vesicles (d = 0.5). Obvious components that are expected to lower the permeability of the plasma membrane are ergosterol and lipids with long and saturated acyl chains as present in sphingolipids (59), but the presence of small molecules partitioning in the hydrophobic core of the membrane should also be considered in future work (60).

## Author Contributions

M.G. and B.P. designed the studies and wrote the manuscript. M.G. and J.F. performed the stopped-flow measurements and analyzed the data. J.v.K., R.H., and L.S. contributed to the strain engineering and in vivo analyses. R.M. and A.J.A.v.M. constructed the yeast manifold knockout strain and assisted in the design and analysis of the in vivo measurements. B.P. supervised the work.

## Figures and Tables

**Table A1:** Parameters for the Calculation of Relaxation Dynamics Curves

Parameter	Symbol	Value	Unit	Reference
Dissociation constant KPi (in vitro)	pK_1_	7.21	N/A	https://pubchem.ncbi.nlm.nih.gov/
Dissociation constant KPi (in yeast)	pK_2_	6.59	N/A	(30)
Dissociation constant benzoic acid	pK_2_	4.19	N/A	https://pubchem.ncbi.nlm.nih.gov/
Dissociation constant acetic acid	pK_2_	4.76	N/A	https://pubchem.ncbi.nlm.nih.gov/
Dissociation constant formic acid	pK_2_	3.75	N/A	https://pubchem.ncbi.nlm.nih.gov/
Dissociation constant lactic acid	pK_2_	3.86	N/A	https://pubchem.ncbi.nlm.nih.gov/
Dissociation constant pyruvic acid	pK_2_	2.45	N/A	https://pubchem.ncbi.nlm.nih.gov/
KPi deprotonation rate constant	k_1_	10^6^	s^−1^	(61)
Acid deprotonation rate constant	k_2_	10^6^	s^−1^	(61)
Water molar volume	M_1_	18	cm^3^/mol	N/A
Volume of yeast before the osmotic shock	V_0_	81.9	fl	after (26)
Zero-turgor volume	V_r_	66.6	fl	(26)
Nonosmotic volume	b	43.3	fl	(26)
Volumetric elastic modulus	*ε*	4.53	MPa	after (31)
Small solute concentration in yeast cytosol	c_s_	370	mM	after (27)
CO_2_ permeability in yeast	P_CO2_	10^−3^	cm/s	after (29)
Water permeability in yeast	P_1_	0.8	cm/s	(32)

N/A, not applicable.

## Appendix: Parameter List

The following parameters were used for calculation of the relaxation dynamics curves.

## Supporting Material

Document S1. Figs. S1–S15 and Tables S1–S3

Document S2. Article plus Supporting Material
