# Supplementary material for: Weak Acid Permeation in Synthetic Lipid Vesicles and Across the Yeast Plasma Membrane
Source: Biophys J. 2019 Nov 27;118(2):422–34. doi: 10.1016/j.bpj.2019.11.3384 (PMC6976801; doi:10.1016/j.bpj.2019.11.3384)
Supplement: Document S1. Figs. S1–S15 and Tables S1–S3 [file mmc1.pdf]

Biophysical Journal, Volume *118*

Supplemental Information

Weak Acid Permeation in Synthetic Lipid Vesicles and Across the Yeast Plasma Membrane

Matteo Gabba, Jacopo Frallicciardi, Joury van 't Klooster, Ryan Henderson, Łukasz Syga, Robert Mans, Antonius J.A. van Maris, and Bert Poolman

Supplementary Figures

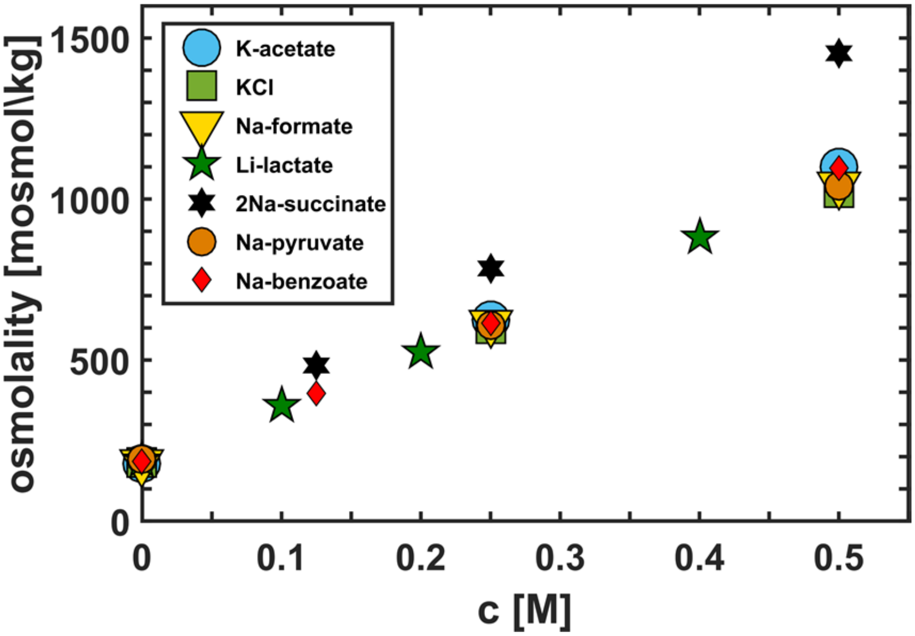

Figure S1: Plots of the measured osmolality as a function of the osmolyte concentration (c). The data were fitted with a linear relation (y = mx + q) that was later used to prepare the osmolyte solutions at the desired osmolality of ca. 300 mosmol/kg. The following slopes (m) were used for the calculations: 1840 (K-acetate), 1672 (KCl), 1722 (Na-formate), 1737 (Li-lactate), 2380 (Na_2-succinate), 1694 (Na-pyruvate) and 1712 (Na-benzoate). The intercept was q = 188.

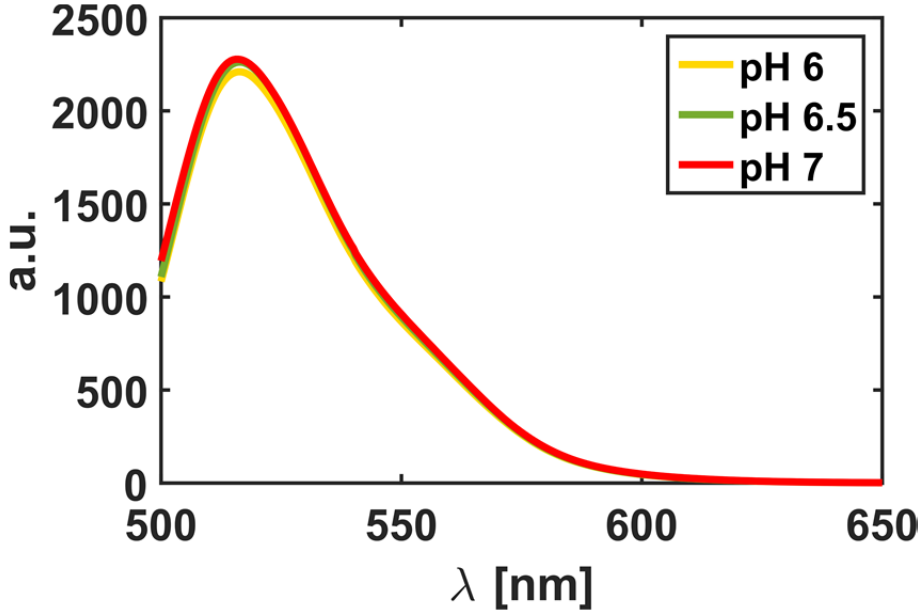

Figure S2: Fluorescence emission spectra of calcein (10 µM) in 100 mM KPi at pH 7.0, 6.5, and 6.0. Spectra were collected at 20°C upon excitation at 495 nm, i.e. the same wavelength as used for the stopped-flow measurements. The bandwidths were set to 1 nm (excitation) and 5 nm (emission), respectively.

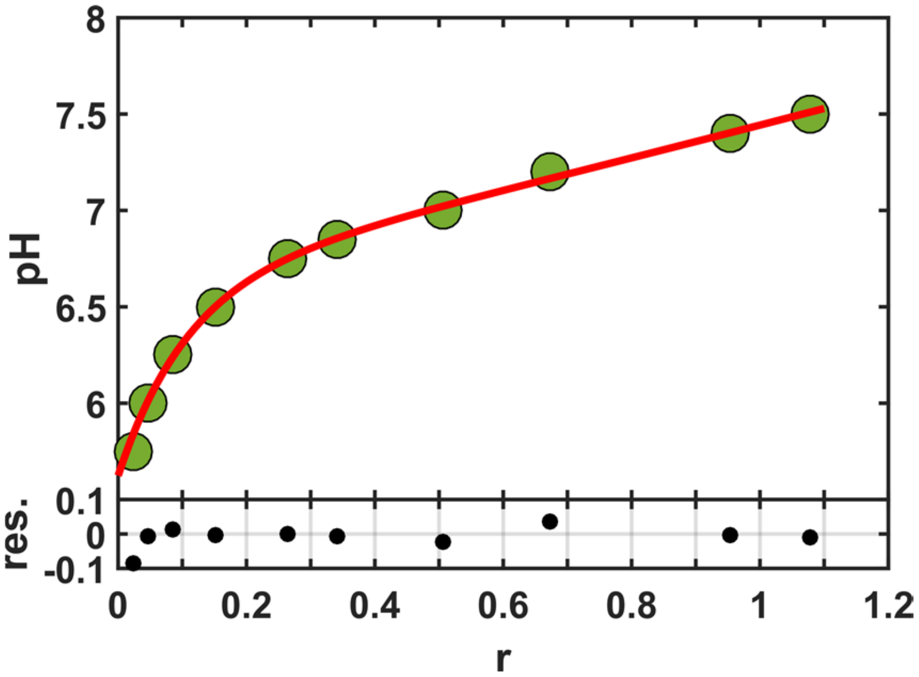

Figure S3: The measured ratio (r = F_{453}/F_{405}) of the blank-subtracted fluorescence intensities of pyranine upon excitation at 453 nm and 405 nm as a function of the buffer pH. The red line is an empirical bi-exponential fit (pH = a*exp(b*r) + c*exp(d*r), which was used to convert the measured ratios to pH values. The parameters are: a = 6.633, b = 0.1152, c = -1.009, and d = -9.241. The residuals (res.) are given in the bottom panel.

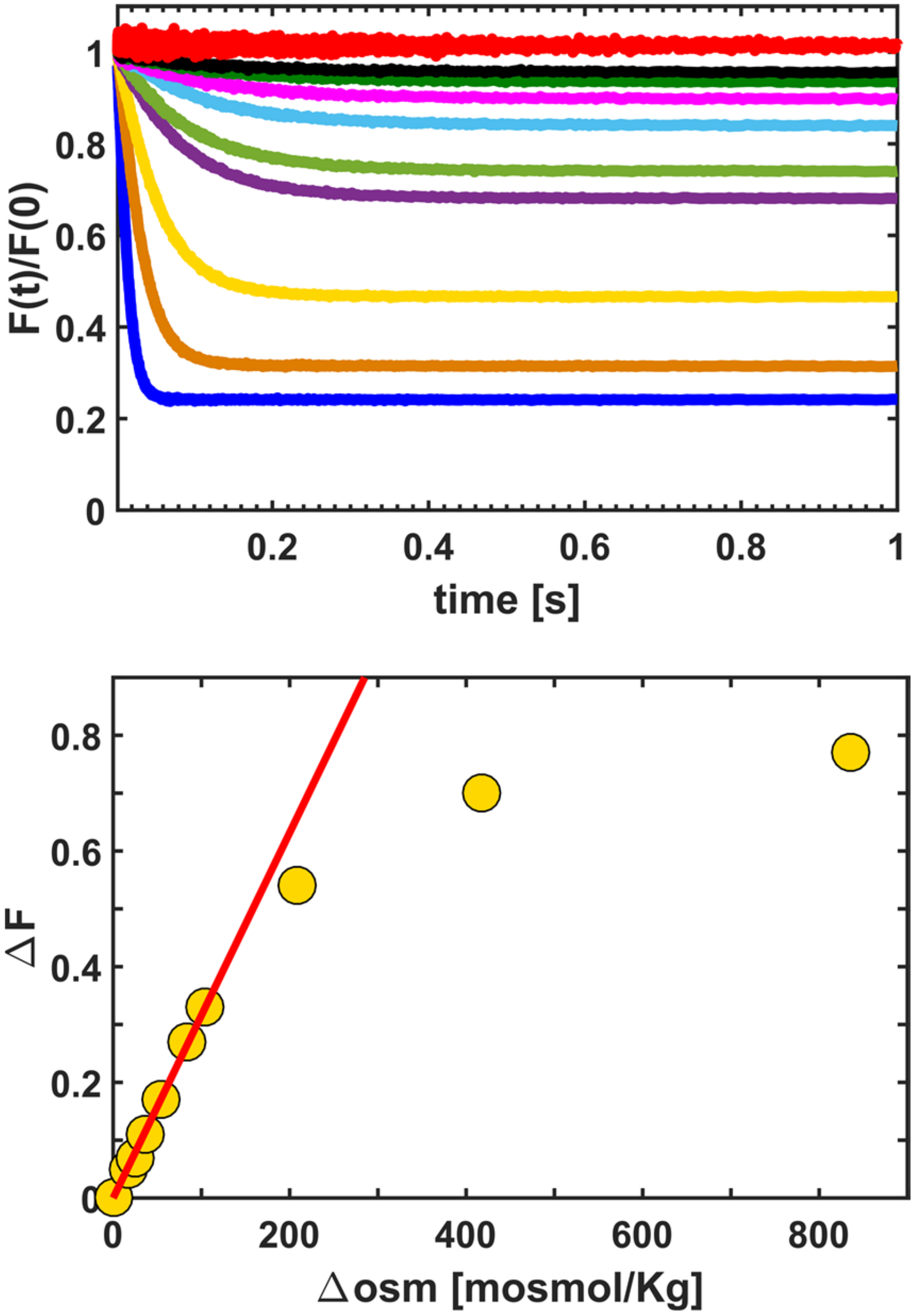

Figure S4: *Upper panel:* Calcein fluorescence development upon osmotic upshift (addition of KCl) of liposomes as measured on the stopped-flow apparatus. The liposomes were prepared in 100 mM KPi pH 7.0 as described in the Materials and Methods section. From top to bottom the KCl concentrations are: 0M, 10 mM, 15.2 mM, 21.5 mM, 32.5 mM, 49.5 mM, 62.5 mM, 125 mM, 250 mM and 500 mM. The liposomes were composed of DOPE:DOPG:DOPC at a weight ratio of 2:1:1. *Bottom panel*: Fluorescence intensity variation before and after the osmotic upshift: $\Delta F = (F_{unshocked} - F_{shocked})/F_{(0)}$ as a function of the applied osmotic gradient (□osm = $osm_{out} - osm_{in}$). The $F_{unshocked}$ and $F_{shocked}$ values are obtained from the plots shown in the upper panel. The intensity variation is linear up to ca. 120 mosmol/kg.

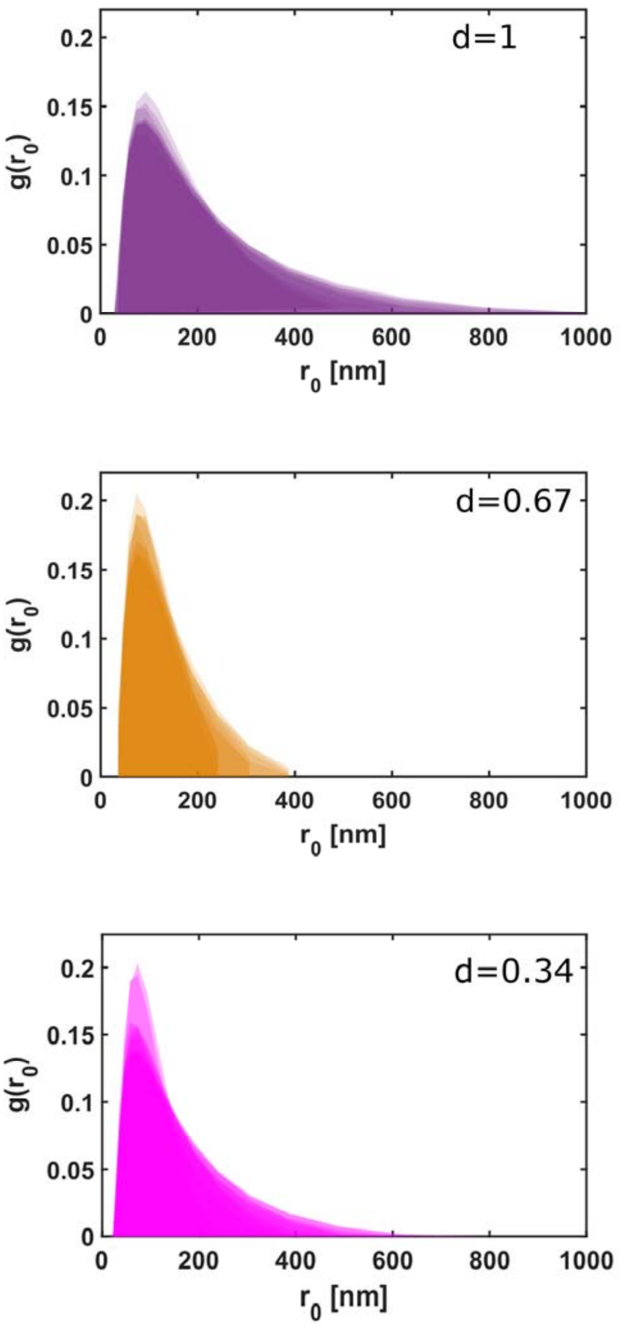

Figure S5: Size distributions of vesicles measured with dynamic light scattering (DLS). The degree of unsaturation d of each mixture is indicated in the panels. For each mixture, ten distributions were measured. To obtain permeability coefficients for water and the weak acids, each of the ten distributions was used to fit the calcein kinetic data with the mathematical model described in the accompanying paper.

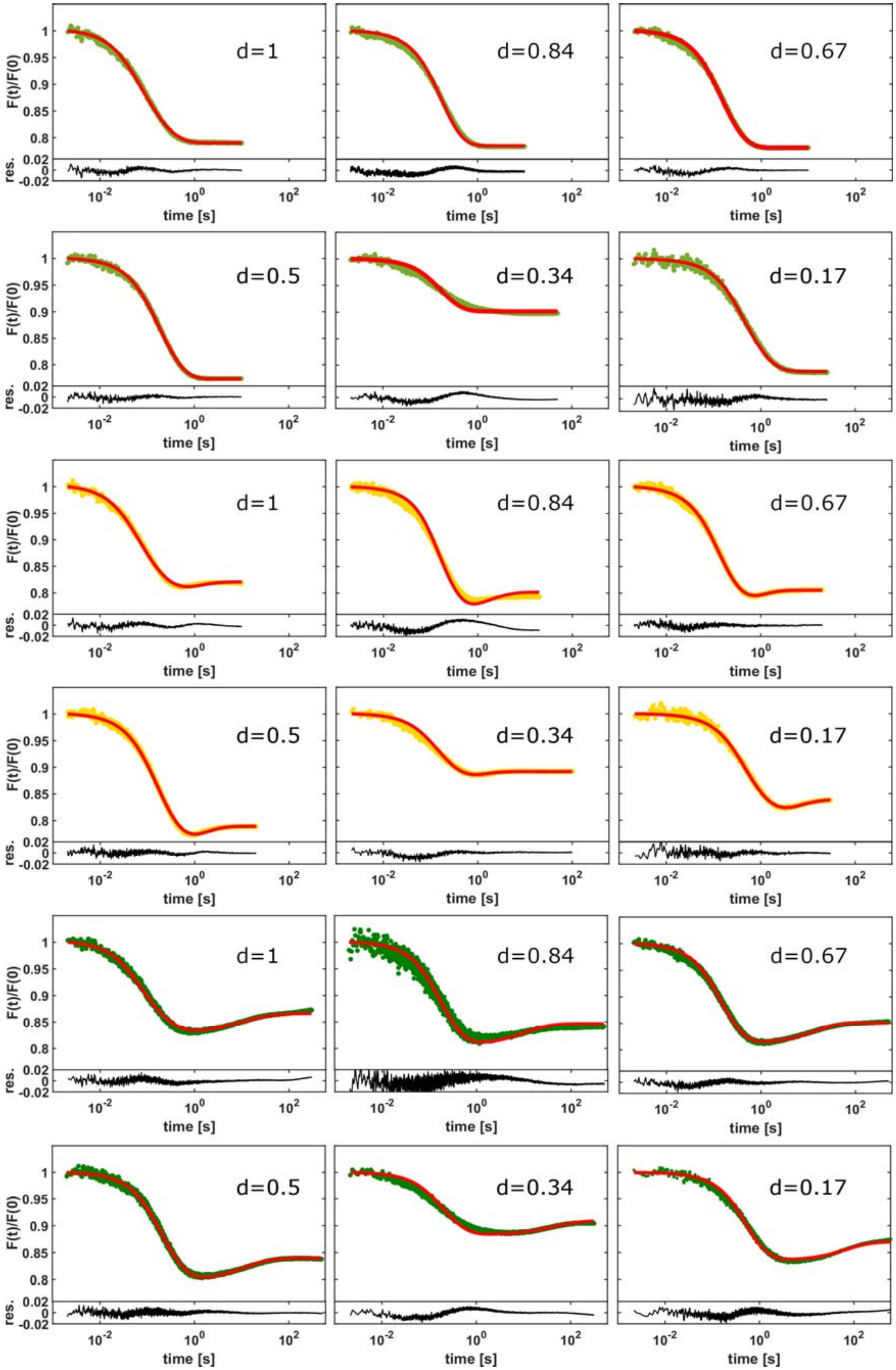

Figure S6: Fits of the calcein relaxation curves for different d values. Osmotic upshift with KCl in light green, with Na-formate in yellow, and with Na-Lactate in dark green. Permeability coefficients of water, formic acid and lactic acid obtained by fitting are presented in Supplementary Table S2.

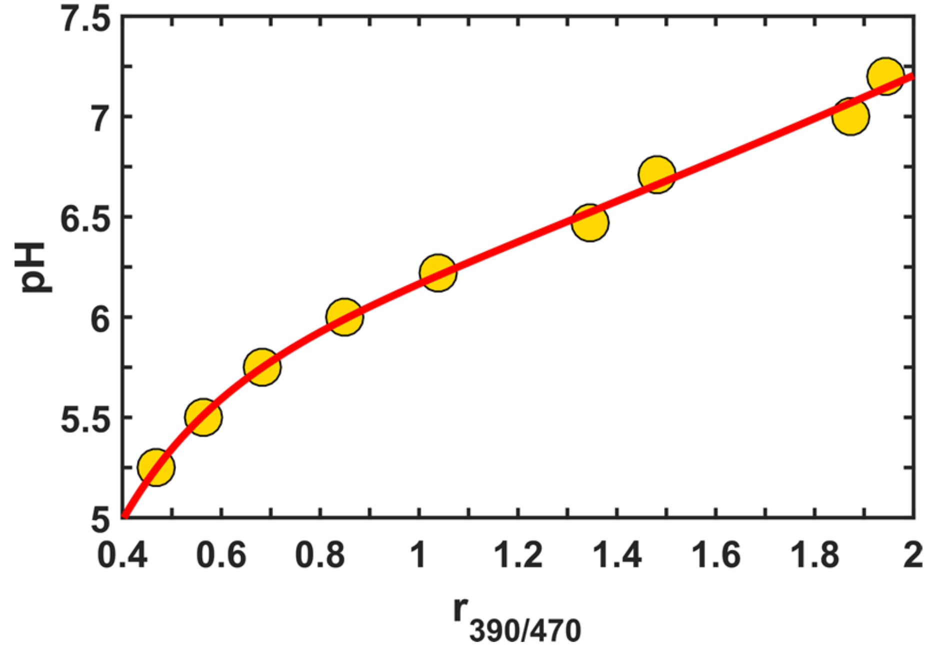

Figure S7: pHluorine calibration curve measured *in vivo,* that is, in *S. cerevisiae* RA380. Fluorescence intensities were recorded with the fluorometer for 90 seconds at 512 nm in dual-wavelength excitation mode (λ_{ex} = 390 nm and 470 nm). The ratio $r_{390/470}$ between the time-averaged intensities was calculated and plotted as a function of the pH. The data set was fitted with a bi-exponential function: pH = a*exp(b*r) + c*exp(d*r). The fitting parameters are: a = 5.33, b = 0.1507, c = -5.195 and d = -5.109.

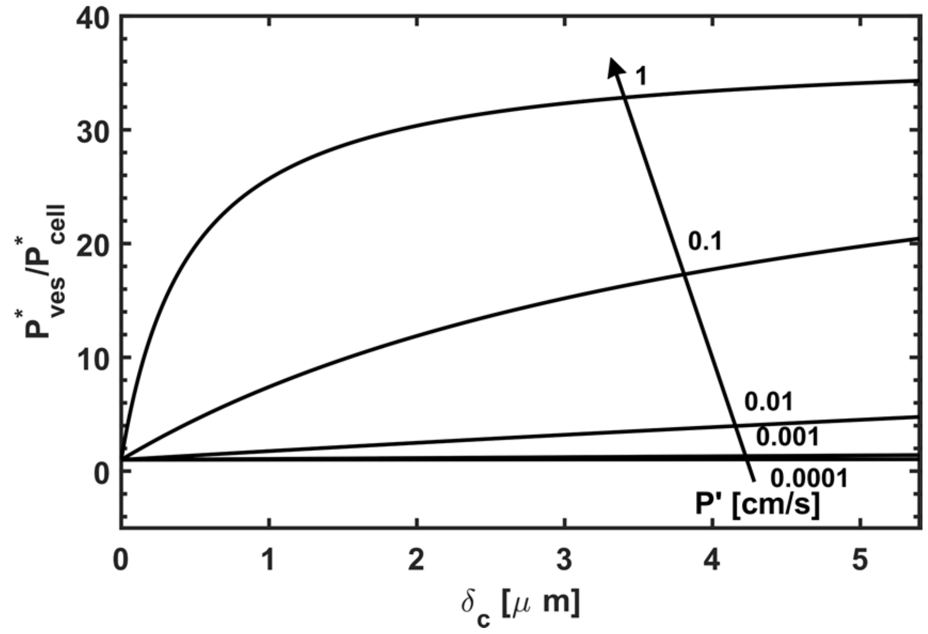

Figure S8: Estimated ratio P_v/P_c between apparent permeability coefficients of vesicle (P_v) and yeast (P_c) as a function of the unstirred layer thickness δ_c and the actual permeability coefficient $P'=P'_v=P'_c$, which was varied from 1 to 10^{-4} cm/s as indicated in the figure. The cell and vesicle diameters were set to 5.4 μm and 0.25 μm, respectively, and the diffusion coefficient D_v of the permeants (acetic and formic acid) *in vitro* to10^{-5} cm^2/s. The diffusion coefficient in the cytosol was set to $D_c = D_v/8$.

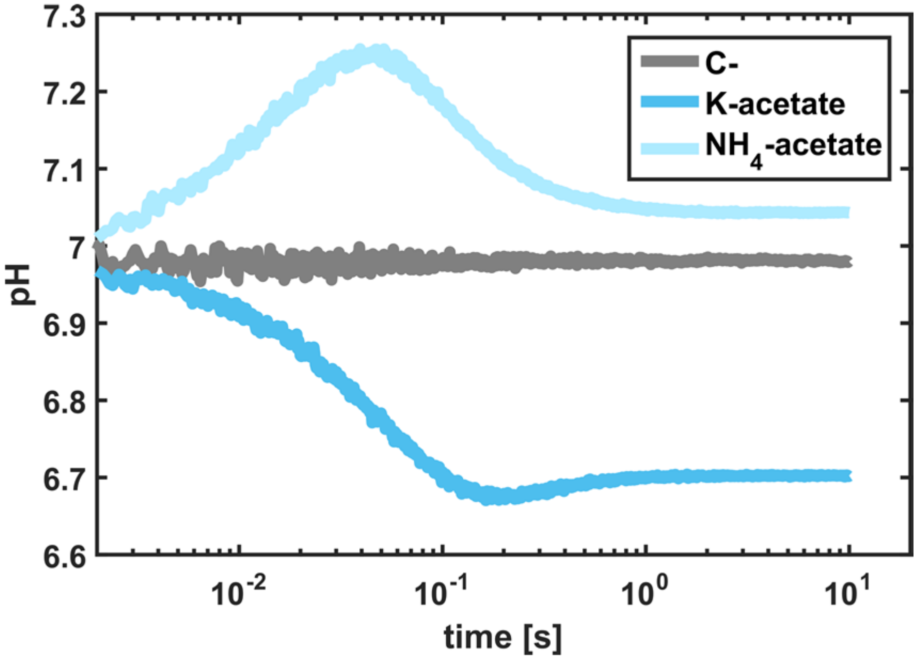

Figure S9: pH-kinetic data obtained with the pyranine assay using liposomes composed of POPE:POPG:POPC at a 2:1:1 weight ratio.

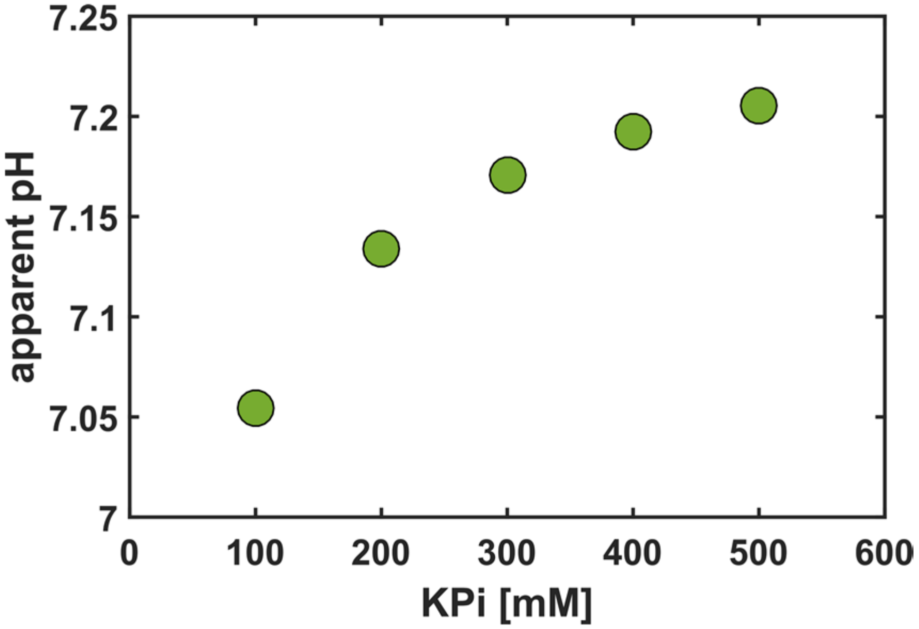

Figure S10: Apparent pH measured with pyranine in KPi of different molarities at pH 7.05 ± 0.03. The plot shows that the pyranine readout depends on the ionic strength of the solution.

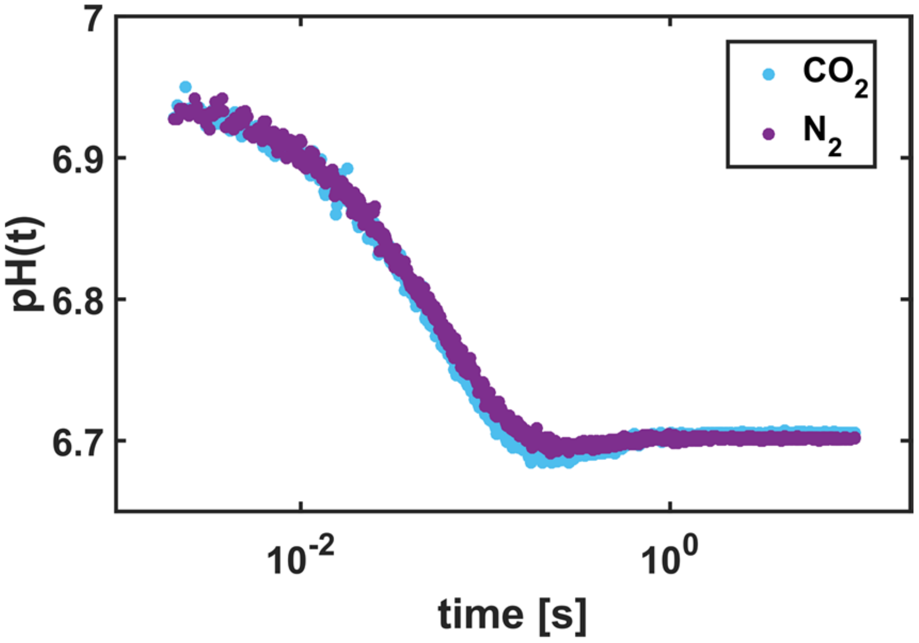

Figure S11: Comparison of pH kinetics measured in 100 mM KPi solutions saturated with CO_2 (light blue dots) or N_2 (violet dots) upon osmotic upshift with K-acetate, using vesicles with the following lipid composition: POPE:POPC:POPG (2:1:1). The solutions were bubbled for 60 min with pure N_2 or CO_2 to saturate them with the gasses.

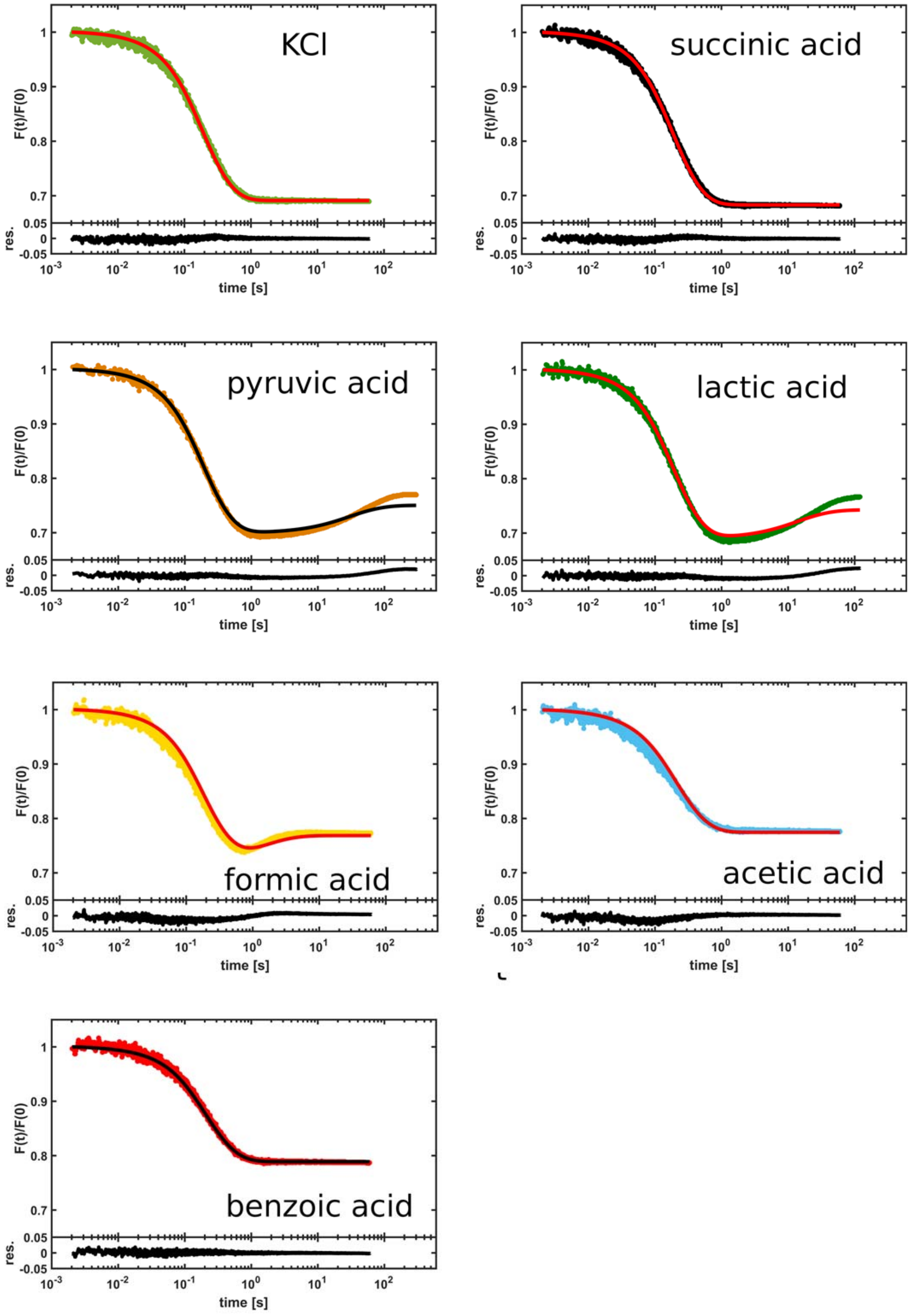

Figure S12: Fits of the calcein relaxation curves with the theoretical model, described in the accompanying paper. The fitting parameters are presented in Supplementary Table S1.

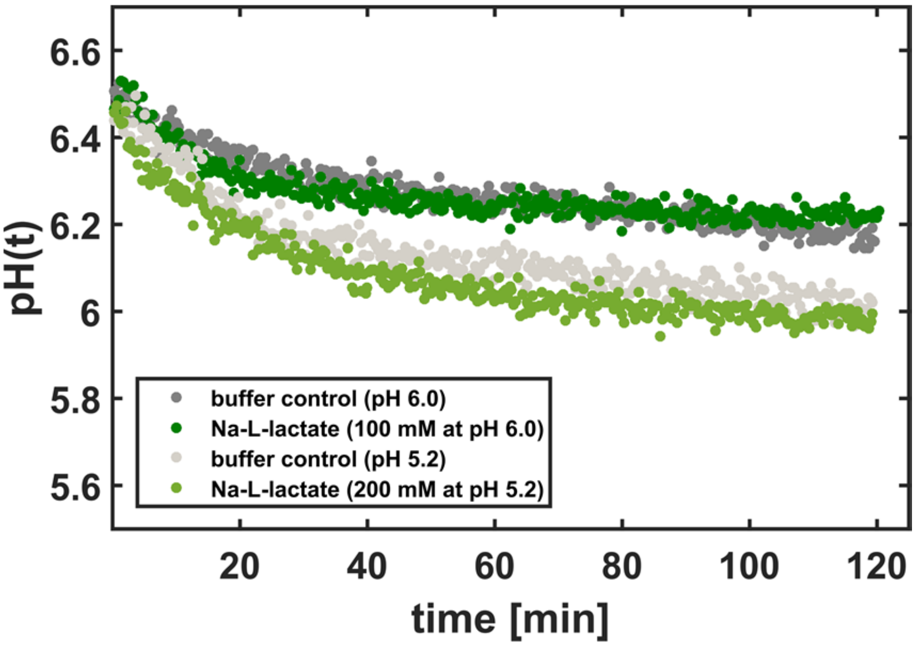

Figure S13: Comparison of *in vivo* pH-kinetics measured with Na-L-lactate at different pH-values (6.0 and 5.2) and concentrations (100 mM and 200 mM), using the *S. cerevisiae* RA380 strain. No significant variations with respect to the buffer control are observed.

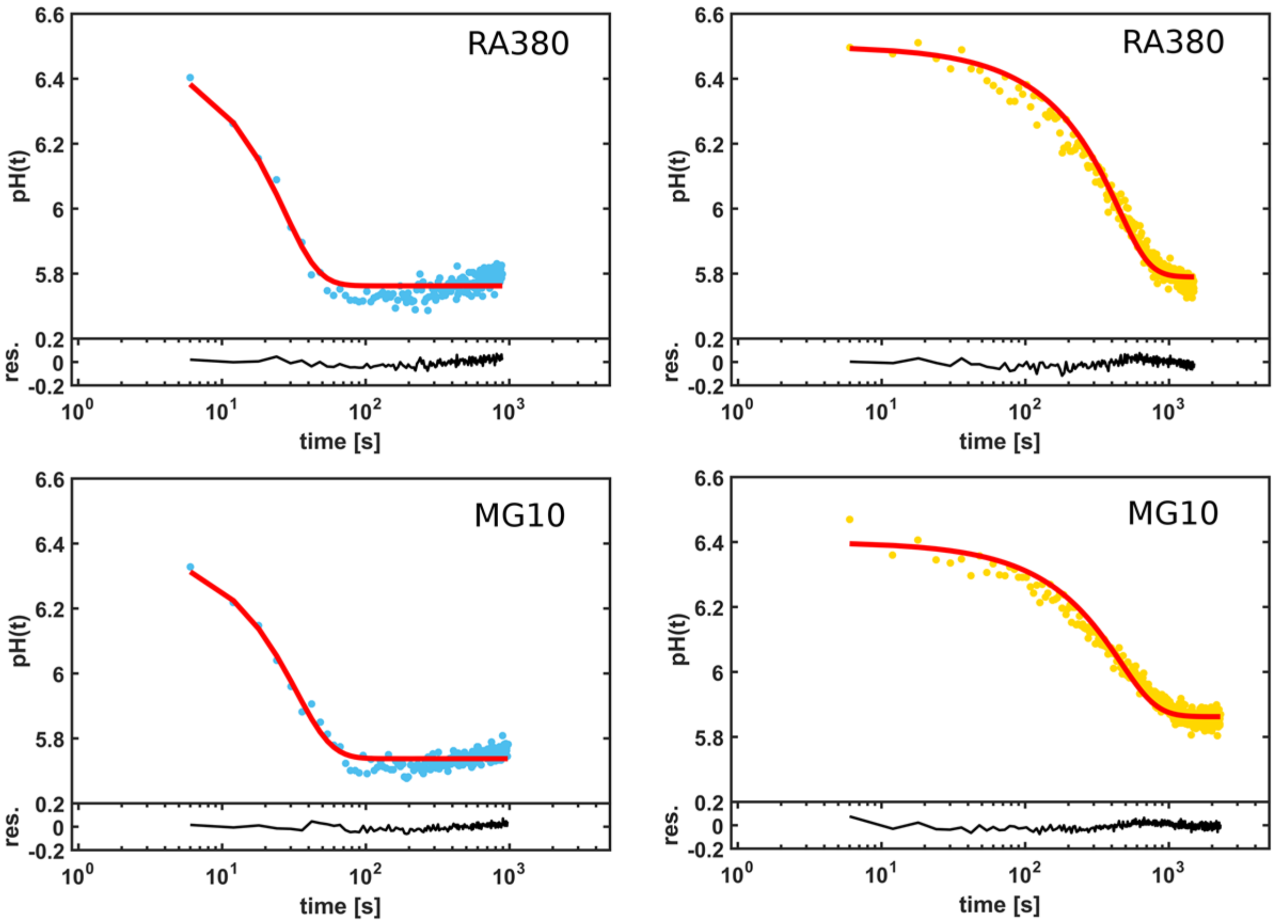

Figure S14: Fits of the pHluorine relaxation curves measured with the fluorometer *in vivo* (osmotic upshift with K-acetate in blue and with Na-formate in yellow), using the theoretical model described in the accompanying paper. The fitting parameters are presented in Supplementary Table S3.

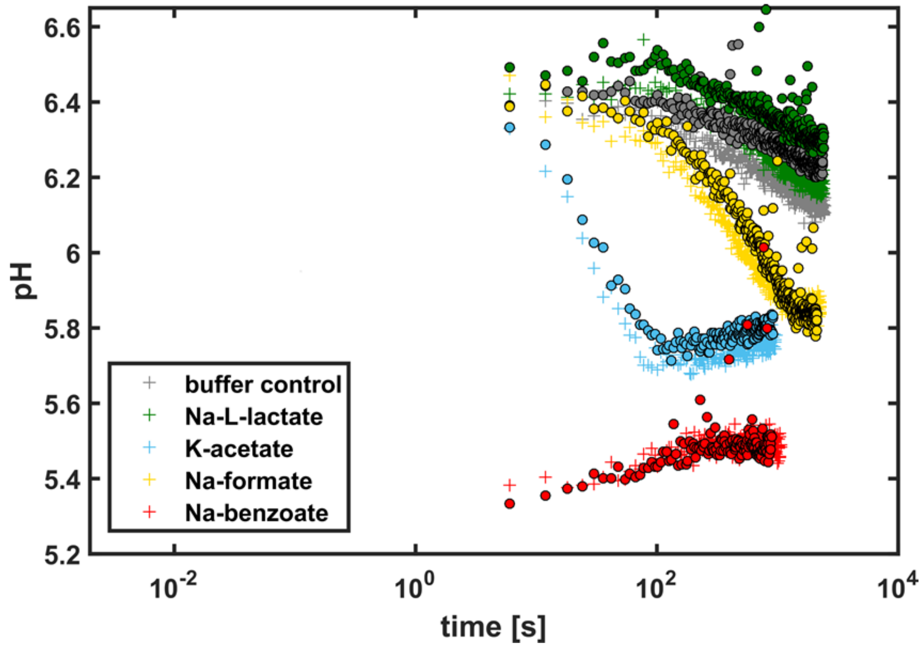

Figure S15: Kinetics of weak acid diffusion measured *in vivo* with the fluorometer in the *S. cerevisiae* MG10 (cross) and Y7001 (circle).

Supplementary Table 1:

	K_D (M^{-1})	P_w (10^{-3}) (cm/s)	P_{AH} (10^{-3}) (cm/s)
Na_2-succinate	51	5.2	/
KCl	49	5.3	/
Na-pyruvate	53	/	0.9
Li-lactate	43	/	0.06
Na-formate	46	/	2.1
K-acetate	37	/	9.9
Na-benzoate	50	/	100

Table S1: Parameters obtained by fit of the calcein kinetics, using the theoretical model described in the accompanying paper; the data were obtained with liposomes prepared from POPE:POPG:POPC lipids at a 2:1:1 weight ratio.

Supplementary Table 2:

Lipid mixture	d	P_{water} *10^{-3} (cm/s)	P_{formic} *10^{-3} (cm/s)	P_{lactic} *10^{-3} (cm/s)
DOXX	1	15±1	7.4±0.9	0.12±0.04
DOXX/POXX	0.84	8.3±0.5	4.9±0.5	0.10±0.01
DOXX/POXX	0.67	6.5±0.5	3.6±0.7	0.07±0.01
POXX	0.5	5.2±0.4	2.1±0.1	0.06±0.01
POXX/DPXX	0.34	4.7±0.4	1.3±0.1	0.026±0.004
DPXX/POXX	0.17	4.2±0.4	0.53±0.07	0.016±0.002

Table S2: Permeability coefficients of water, formic acid and lactic acid as a function of the degree of saturation d, which is indicated in the first column.

Supplementary Table 3:

Osmolyte	Strain	[KPi] (mM)	P (10^{-5}) (cm/s)	$P_{vesicles}/P_{yeast}$
Na-formate	RA380	186	1.1±0.1	192
	MG10	318	1.2	175
K-acetate	RA380	181	1.4±0.2	707
	MG10	160	1.2	825

Table S3: Parameters obtained by fitting the pH kinetics of *S. cerevisiae* RA380 and MG10, using the theoretical model (see Materials and Methods). The fluorometer data were used for the fits (see Supplementary Figure S14). The fold difference between the permeability coefficients obtained in vesicles and the values measured in yeast (see Table S1) is reported in the last column.
